# *FoxB*, a new and highly conserved key factor in arthropod dorsal–ventral (DV) limb patterning

**DOI:** 10.1186/s13227-019-0141-6

**Published:** 2019-11-08

**Authors:** Miriam Heingård, Natascha Turetzek, Nikola-Michael Prpic, Ralf Janssen

**Affiliations:** 1 Department of Earth Sciences, Palaeobiology, Uppsala University, Villavägen 16, Uppsala, Sweden; 2 Abteilung für Entwicklungsbiologie, Johann-Friedrich-Blumenbach-Institut für Zoologie und Anthropologie, Georg-August-Universität, Göttingen, Germany; 3 Present Address: Göttingen Center for Molecular Biosciences (GZMB), Ernst-Caspari-Haus, Göttingen, Germany; 4 Present Address: Department of Geology, Faculty of Science, Lund University, Sölvegatan 12, Lund, Sweden; 5 Present Address: Bereich Allgemeine Zoologie und Entwicklungsbiologie, Institut für Allgemeine und Spezielle Zoologie, Justus-Liebig-Universität Gießen, Heinrich-Buff-Ring 38, 35392 Gießen, Germany

**Keywords:** Appendage patterning, Forkhead domain, Limb segmentation, Development

## Abstract

Forkhead box (Fox) transcription factors evolved early in animal evolution and represent important components of conserved gene regulatory networks (GRNs) during animal development. Most of the researches concerning Fox genes, however, are on vertebrates and only a relatively low number of studies investigate Fox gene function in invertebrates. In addition to this shortcoming, the focus of attention is often restricted to a few well-characterized Fox genes such as *FoxA* (*forkhead*), *FoxC* (*crocodile*) and *FoxQ2*. Although arthropods represent the largest and most diverse animal group, most other Fox genes have not been investigated in detail, not even in the arthropod model species *Drosophila melanogaster*. In a general gene expression pattern screen for panarthropod Fox genes including the red flour beetle *Tribolium castaneum*, the pill millipede *Glomeris marginata*, the common house spider *Parasteatoda tepidariorum*, and the velvet worm *Euperipatoides kanangrensis*, we identified a Fox gene with a highly conserved expression pattern along the ventral ectoderm of arthropod and onychophoran limbs. Functional investigation of *FoxB* in *Parasteatoda* reveals a hitherto unrecognized important function of FoxB upstream of *wingless* (*wg*) and *decapentaplegic* (*dpp*) in the GRN orchestrating dorsal–ventral limb patterning.

## Introduction

Arthropod limbs develop along three different axes, the proximal–distal (PD) axis, the anterior–posterior (AP) axis, and the dorsal–ventral (DV) axis. In the model system *Drosophila melanogaster*, leg allocation and AP axis determination is under control of segment polarity genes such as *wingless* (*wg*) and *hedgehog* (*hh*) (e.g. [1, 2]). This is likely conserved in arthropods and onychophorans as indicated by gene expression and functional data (e.g., [3–10]). The PD axis is established by the function of the so-called limb gap genes and the morphogens Wg and Decapentaplegic (Dpp) (e.g., [1, 11–13]), and gene expression data suggest that the function of limb gap genes is generally conserved among arthropods and onychophorans (e.g., [3, 10, 14–19]). In *Drosophila*, the morphogens Dpp and Wg are also involved in the determination of the DV axis [20–22]. The *wg* gene is expressed in the ventral region of the leg imaginal discs and loss of Wg protein causes dorsalisation of these limbs [22–24]. Downstream of Wg act two T-box genes, the paralogs *H15* and *midline* (*mid*), both of which are like *wg* expressed in ventral ectodermal cells of the limbs [4, 20, 25–27]. *dpp* is expressed along the dorsal side of the *Drosophila* leg imaginal disc and loss of Dpp causes ventralization of these limbs [28, 29]. Downstream of Dpp functions another T-box gene, *optomotor*-*blind* (*omb*), which is expressed along the dorsal side of the legs [20, 29]. Expression of *omb* can induce dorsal fate in ventral cells of the developing legs [29]. Comparative gene expression data suggest that the role of ventral and dorsal leg patterning genes is conserved in arthropods, and partially also in onychophorans (e.g., [10, 26, 30–32]). However, functional evidence of a conserved DV patterning system in arthropods is sparse and exclusively based on data in insects [33, 7, 9, 25, 34]. While *wg* appears to be involved in DV limb development in holometabolous insects [7, 9], this does not appear to be the case for hemimetabolous insects [33].

Notably, many of the genes involved in DV limb patterning are duplicated in *Drosophila*, as well as in other studied arthropods and onychophorans. There are two *H15*-type genes in *Drosophila* and the millipede *Glomeris marginata*, and at least three *H15* genes in spiders, and two copies of *omb* in onychophorans and spiders (summarized in [32]). Many of these genes have retained conserved expression patterns along the ventral and dorsal region of the developing limbs, respectively. Therefore, it is likely that their function(s) in DV patterning are at least partially conserved as well. This makes functional studies difficult because of likely redundant functions of such paralogs. Although *wg* is not duplicated in arthropods and onychophorans [6, 35, 36], it is a member of the Wnt class of genes (*wg* is *Wnt1*) of which arthropods ancestrally possess 12 classes (reviewed in [36, 37]). Many Wnt genes, although not paralogs of *wg*/*Wnt1*, are expressed in very similar patterns along the ventral side of the developing arthropod limbs (e.g., [38, 36]). It is therefore possible that other Wnt genes may substitute for *wg* function, and that functional data on the role of *wg* in arthropod limb development are inconclusive and potentially misleading (cf. [7, 9, 33]).

Here we report on the discovery of a hitherto unrecognized gene that is expressed along the ventral side of the investigated arthropods and an onychophoran, the forkhead transcription factor-encoding gene *FoxB* (*Drosophila* paralogs *Dmfd4*/*Dmfd5* aka *fd96Ca*/*fd96Cb* [39, 40]. Although it exists in two copies (paralogs) in the model arthropods *Drosophila* and the flour beetle *Tribolium castaneum*, there is only one copy in the spider *Parasteatoda tepidariorum*. We therefore targeted the spider *FoxB* gene (*Pt*-*FoxB*) in our study and investigated its function in appendage development. Among other phenotypes, *Pt*-*FoxB* knockdown leads to altered leg morphologies, likely correlated with disturbed DV patterning during limb development. The expression of other known (or implied by conserved expression patterns) DV limb patterning genes such as *omb*, *H15*, and *wg*/*Wnt1* is disturbed in *Pt*-*FoxB* knockdown appendages. This indicates a high-ranking function of *FoxB* in the gene regulatory network orchestrating DV limb patterning in spiders as well as Panarthropoda as a whole.

## Methods

### Research animals, embryo collection and developmental staging

*Drosophila* flies and embryos were obtained from the cultures in Göttingen (*Oregon*-*R strain*). *Tribolium* embryos were obtained from the cultures in Göttingen (*San Bernardino strain*). *Glomeris* embryos were collected and prepared as described in Janssen et al. [7]. *Parasteatoda* spiders were obtained from the established *Göttingen strain* for RNA-interference experiments. They were kept separately in plastic vials at approximately 21 °C. They were supplied with water and fed with either sub-adult crickets (*Acheta domesticus*) or *Drosophila*. *Euperipatoides kanangrensis* embryos were obtained as described in Hogvall et al. [41]. Developmental staging is after Janssen et al. [8] (*Glomeris*), Janssen and Budd [42] (*Euperipatoides*), Mittmann and Wolff [43] (*Parasteatoda*), and Strobl and Stelzer [44] (*Tribolium*).

### Gene cloning, probe synthesis, whole mount in situ hybridization and nuclear staining

All gene fragments were isolated by means of RT-PCR with gene-specific primers based on sequence information from either sequenced genomes (*Tribolium*, Tribolium Genome Sequencing Consortium [45] and *Parasteatoda*, Schwager et al. [46]), or sequenced embryonic transcriptomes (*Glomeris* and *Euperipatoides*). Primer sequences are listed in Additional file 1: Table S1. Genes were cloned into either the PCRII vector (Invitrogen) or the PCR4-TOPO vector (Invitrogen). Sequences of the cloned fragments were verified by means of Big Dye chemistry on an ABI3730XL sequence analyser by a commercial sequencing service (Macrogen, Seoul, South Korea). Accession numbers of all gene fragments are listed in Additional file 2: Table S2.

Antisense RNA probes were in vitro transcribed with either Sp6, T7 or T3 RNA Polymerase (Roche) after amplification of the gene products from the plasmids using M13F and M13R oligonucleotides (Additional file 1: Table S1). Embryos of *Tribolium* were fixed as described in Schinko et al. [47]. Embryos of *Parasteatoda* were fixed as described in Pechmann et al. [48], or, after RNAi treatment, were fixed in a formaldehyde–heptane mix (1:15) for 4 h. Thereafter, the vitelline membranes were removed with Dumont-5 forceps. There is no significant difference in the result of in situ hybridization with either of the spider fixation protocols. Embryos of *Glomeris* were fixed in the same way as spider embryos after RNAi treatment. Embryos of *Euperipatoides* were fixed in a 1:15 formaldehyde-PBS mix for 4–6 h; membranes were removed with Dumont-5 forceps prior to fixation. For all single-colour stainings in all embryos, we applied the whole mount in situ hybridization protocol as described in Janssen et al. [49]. Double in situ hybridization was performed as described in Janssen et al. [50] with the exception that BM Purple (blue signal) (Roche) and SIGMAFAST Fast Red TR/Naphthol AS-MX (red signal) (SIGMA) were used for probe detection. Cell nuclei were visualized incubating embryos in 2 μg/ml of the fluorescent dye 4-6-diamidino-2-phenylindole (DAPI) in phosphate-buffered saline with 0.1% Tween-20 (PBST) for 20–30 min.

### dsRNA synthesis and parental RNAi

After amplification from the plasmid using T7 and T7-Sp6 overhang primers (Additional file 1: Table S1), double-stranded RNA (dsRNA) for *Pt*-*FoxB* was synthesized using the MEGAscript T7 Kit (Life technologies). Sodium acetate was used for precipitation and RNA was dissolved in injection buffer (1.4 mM NaCl, 0.07 mM Na_2_HPO_4_, 0.03 mM KH_2_PO_4_, 4.0 mM KCL). Female spiders were injected laterally into the opisthosoma and either mated several hours prior to injection, or several hours after the first injection. We performed two independent rounds of injection that differed in the concentration of injected dsRNA. Each spider was injected three times (on 3 consecutive days) with each time 2.5 µl of 2.8 µg/µl dsRNA (first round of injections) or 4 µg/µl dsRNA (second round of injections) in injection buffer. Control spiders were injected with 2.5 μl of injection buffer. Altogether, 20 adult females were injected with *Pt*-*FoxB* dsRNA and 20 females with buffer only. Of all surviving spiders, cocoons of dsRNA-injected spiders were kept in glass vials separately. All dsRNA-injected and all control cocoons, respectively, were pooled and investigated each as one batch (see Additional file 3: Figure S1 for quantification). Embryos with morphologically distinguishable phenotypes were categorized into four distinct classes of which one, Class-I, is of particular interest for this study (see “Results”).

### Phylogenetic analysis

Amino acid sequences of the FoxA, FoxB and FoxC forkhead domains of *Parasteatoda*, *Glomeris*, *Tribolium*, *Euperipatoides* and *Drosophila* were aligned using ClustalX with default parameters in MacVector v12.6.0 (MacVector, Inc., Cary, NC). In our analysis, the forkhead domain of *Drosophila* FoxQ2 serves as an outgroup sequence.

Bayesian phylogenetic analysis was performed with MrBayes [51] using a fixed WAG amino acid substitution model with gamma-distributed rate variation across sites (with four rate categories). Unconstrained exponential prior probability distribution on branch lengths and an exponential prior for the gamma shape parameter for among-site rate variation were applied. Topologies were estimated using 0.5 million generations for the Metropolis-coupled Markov chain Monte Carlo analysis with four chains. The chain-heating temperature was set to 0.2, and the chains were sampled every 200 cycles. 25% of samples were used as burnin. Clade support values were computed with posterior probabilities in MrBayes. See Additional file 4: Figure S2.

### Imaging and image processing

All pictures were taken with a Leica DFC490 digital camera mounted on a Leica dissection microscope (MZ-FLIII). The image processing software Adobe PHOTOSHOP CC 2015 (v. 1.0 for Apple Macintosh) was used for the application of linear corrections of contrast and brightness.

## Results

### Expression of panarthropod FoxB genes in developing appendages

**Fig. 1:**
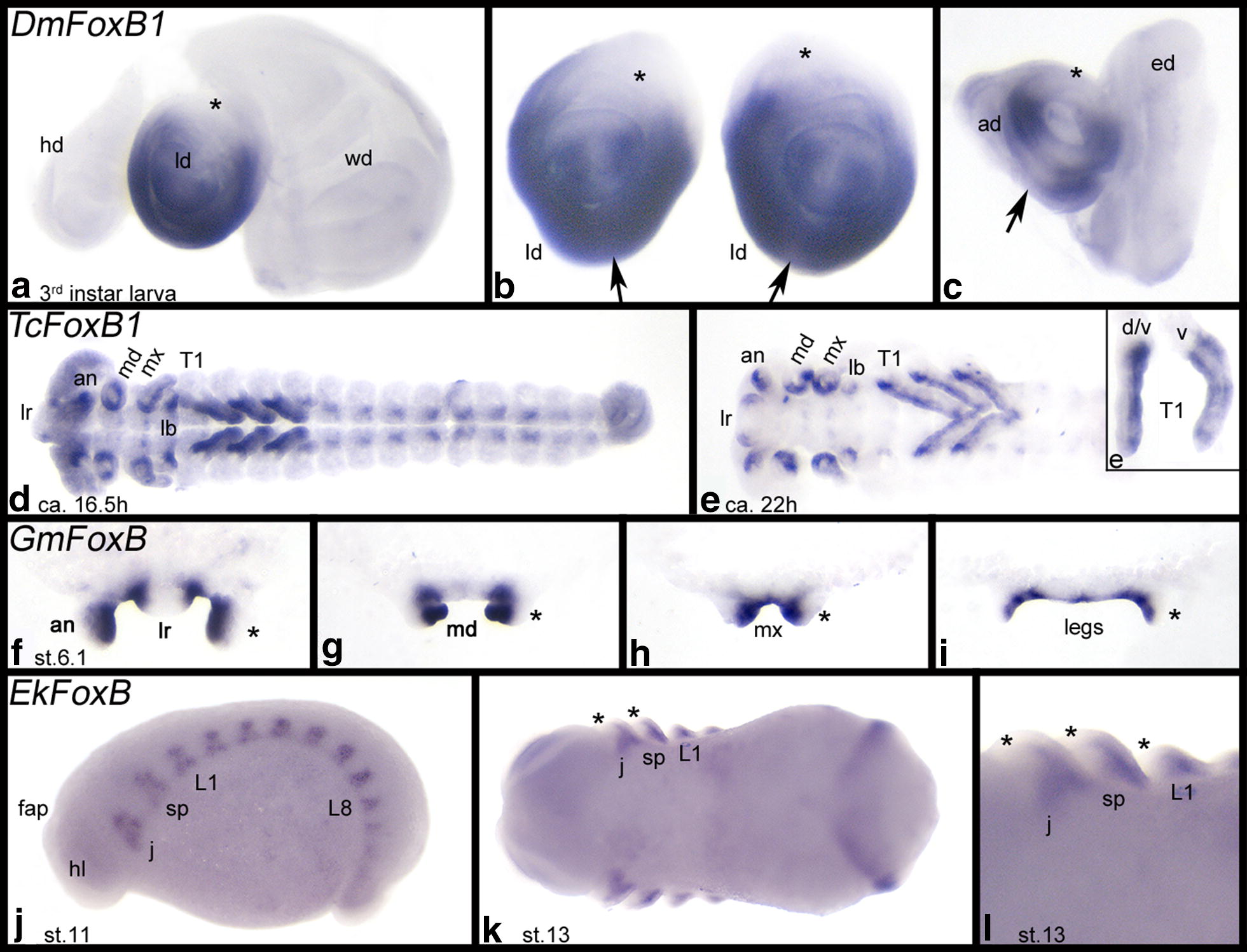
Expression of *FoxB* genes in the developing limbs in *Drosophila melanogaster* (**a–c** dissected imaginal discs), *Tribolium castaneum* (**d**, **e** flat mounted embryos; anterior to the left; ventral view. **e** Inlay shows dissected legs), *Glomeris marginata* (**f**–**i** dissected limbs; anterior view), and *Euperipatoides kanangrensis* (**j**–**l** whole mount embryos; anterior to the left; **j** lateral view; **k**, **l** ventral view). Note that expression is always in the ventral sector of the ventral appendages, except for the arthropod labrum where expression is dorsal. Dorsal appendages, i.e., the wings and halteres in *Drosophila* do not express *FoxB*. Asterisks (*) mark dorsal tissue in ventral appendages. Arrows in **b**, **c** point to weaker (or no) expression in the very ventral region of the leg disc and the antennal disc. *ad* antennal disc, *an* antenna, *ed* eye disc, *fap* frontal appendage, *hd* haltere disc, *hl* head lobe, *j* jaw, *L1/8* first and eighth walking limb, *lb* labium, *ld* leg disc, *lr* labrum, *md* mandible, *mx* maxilla, *sp* slime papilla, *T1* first thorax segment, *v/d* ventral and dorsal side of leg, *v* ventral view on leg, *wd* wing disc

The embryonic expression of *Drosophila FoxB* orthologs [i.e., *Dmfd4*/*Dmfd5* (aka *fd96Ca*/*fd96Cb*)] has been described before [52]. However, in their paper, Häcker et al. [52] only describe embryonic expression patterns, and since appendages develop from imaginal discs in *Drosophila*, expression in the developing limbs remains unclear. We therefore investigated expression in the imaginal discs of third instar larvae. We found that both FoxB orthologs are expressed in identical, albeit weaker for *FoxB2,* patterns in the leg discs and in the antennal discs, but not in the haltere discs, the wing discs, or the eye discs (Fig. 1a–c and Additional file 5: Figure S3). Expression in the leg and antennal discs is restricted to ventral tissue. In both structures, this region of expression is interrupted by a small, most ventral zone of lower (or no) expression.

**Fig. 2:**
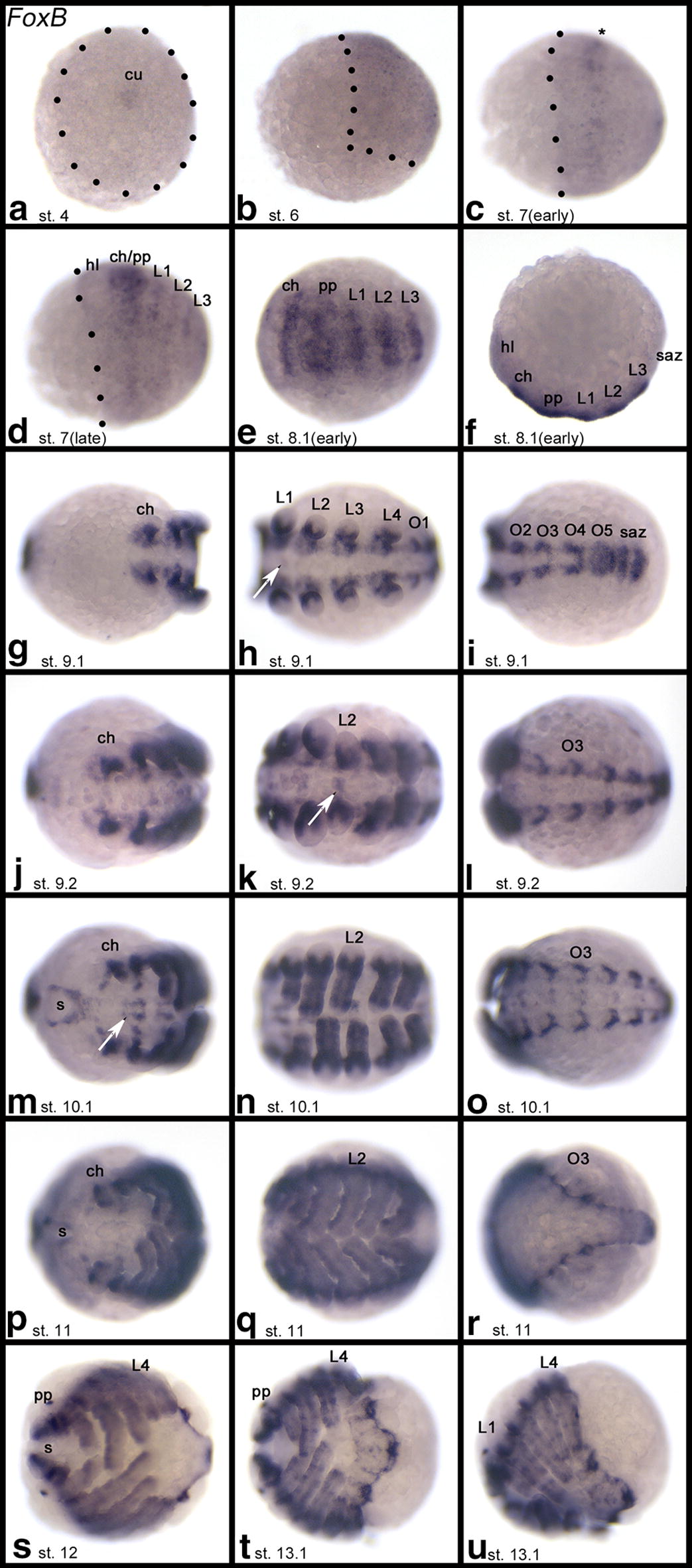
Expression of *Parasteatoda tepidariorum FoxB*. In all panels, anterior is to the left representing ventral views, except **b**, **f**, and **u** (lateral views). Dotted lines in **a**–**d** mark borders of the embryo proper. The asterisk in **c** marks a first stripe of enhanced expression. Arrows in **h**, **k** and **m** point to expression in the ventral nervous system. Embryos of the same stage (**e**–**r**, **t**, **u**) represent different views on the same embryo. *ch* chelicera, *cu* cumulus, *L1–L4* first to fourth walking leg, *O2–O5* second to fifth opisthosomal segment, *pp* pedipalp, *s* stomodaeum, *saz* segment addition zone

**Fig. 3:**
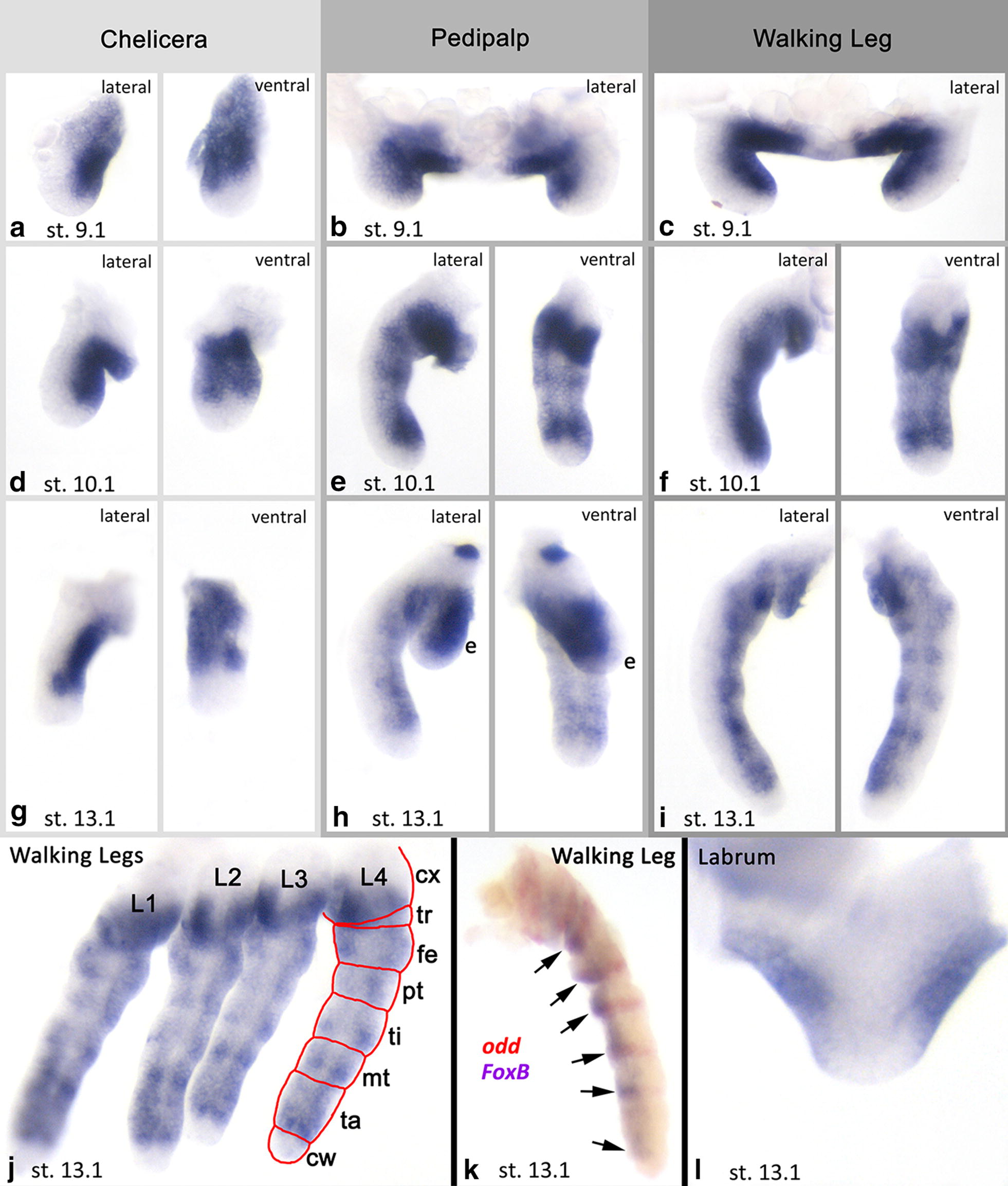
Expression of *Parasteatoda tepidariorum FoxB* and *odd*-*skipped* (*odd*) in dissected limbs. Note that expression of *FoxB* is exclusively in ventral tissue in all investigated stages and appendage types (**a**–**j**) [except for the labrum, **l** where expression is in dorsal tissue. See text for further explanation]. In **a** and **d**–**i**, the left photograph represents a lateral view, the right photograph shows a posterior view on the same appendage. **b**, **c**, **l** Anterior view. **j** Posterior view. **k** Lateral view. Note the appearance of segmental transverse stripes of enhanced expression within the ventral region of *FoxB* expression in the pedipalps and legs (e.g., **e**, **i**, **j**) co-expressed with *odd* (**k**). Arrows in **k** point to co-expression of *odd* and *FoxB*. *cw* claw, *cx* coxa, *e* endite, *fe* femur, *mt* metatarsus, *pt* patella, *ti* tibia, *ta* tarsus, *tr* trochanter

In all three sequentially segmenting arthropods investigated here, i.e., *Tribolium*, *Glomeris* and *Parasteatoda*, *FoxB* genes are expressed in the ventral sector of the developing appendages, except for the labrum where expression is dorsal (and thus in line with the rotation theory [53]) (Figs. 1d–i, 2, 3l and Additional file 6: Figure S4; Additional file 7: Figure S5; Additional file 8: Figure S6). At later developmental stages, expression in the walking limbs refines into a pattern of transverse ventral stripes of stronger expression that likely correspond to the beginning podomerisation (leg segmentation) (Figs. 1e, i and 3). This is most obvious in *Parasteatoda* with its relatively long legs and pedipalps (Fig. 3e–k).

Expression of *FoxB* in the onychophoran *Euperipatoides* (as a panarthropod outgroup species) is restricted to ventral expression early during limb development (Fig. 1j–l). While the slime papillae, the jaws and the walking limbs express *FoxB* in this way, the most anterior appendages, the protocerebral frontal appendages (= the onychophoran antennae) do not express *FoxB* (Fig. 1j). Additionally, FoxB genes are expressed in the developing ventral nervous system in *Drosophila* [51], *Tribolium*, *Glomeris*, *Parasteatoda*, and *Euperipatoides* (Figs. 1 and 2, Additional file 7: Figure S5 and Additional file 8: Figure S6), and in *Glomeris*, *FoxB* is strongly expressed in the anal valves (Additional file 7: Figure S5). At early developmental stages, *Parasteatoda FoxB* is expressed in the centre of the germ disc where the cumulus forms (Fig. 2a), and slightly later when the germ field forms, *FoxB* is expressed ubiquitously (Fig. 2b). When the segments form, *FoxB* is expressed in the form of transverse segmental stripes that later refine to the position of the forming limb buds (Fig. 2c–i).

### FoxB function in the spider *Parasteatoda*

**Fig. 4:**
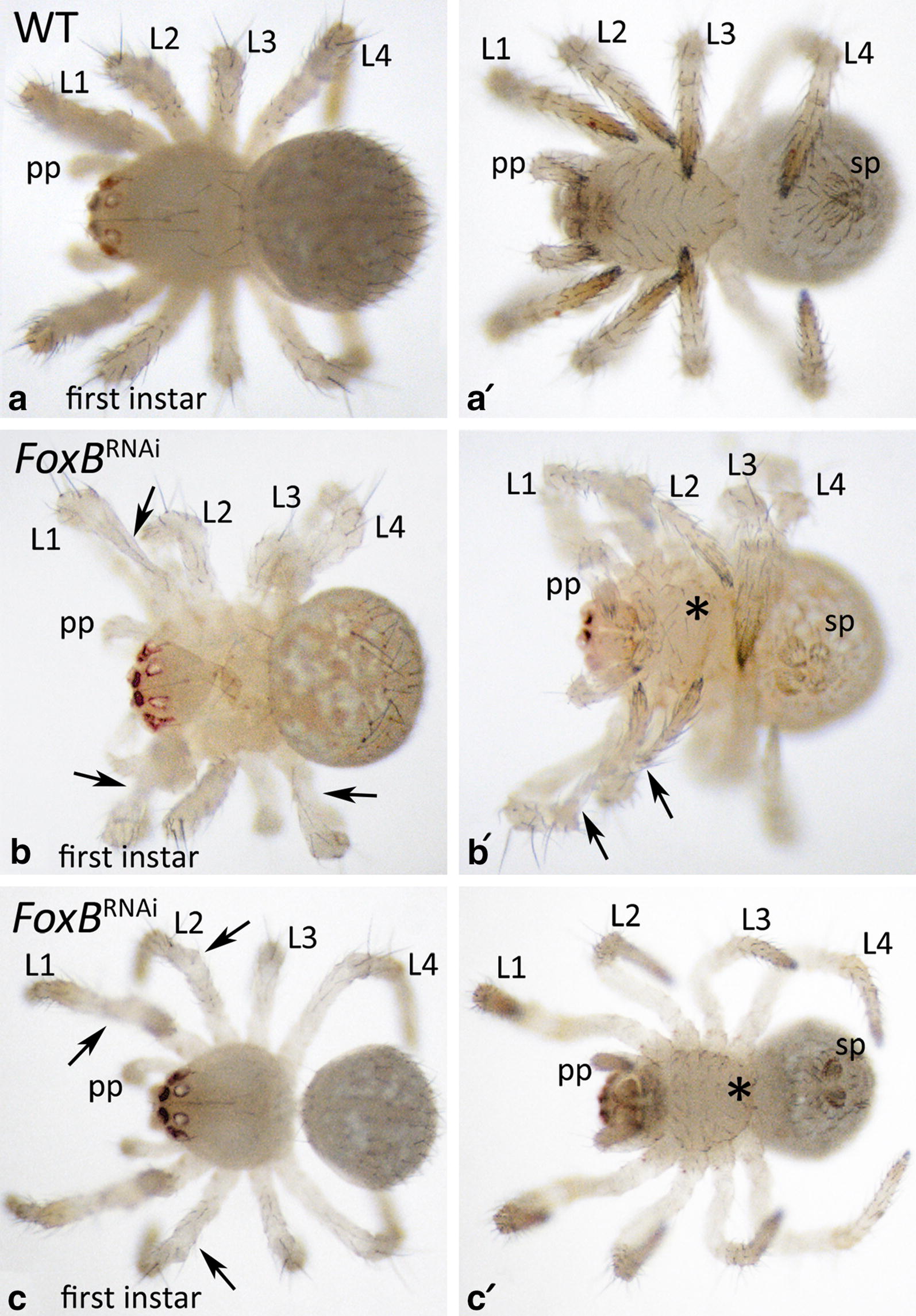
Malformations of the limbs in *FoxB* knockdown first instars. In all panels, anterior is to the left. **a**–**c** Show dorsal views, **a′**–**c′** show the same embryos in ventral views. Arrows point to malformations. Asterisk (*) in **b′** and **c′** mark disturbed ventral bristle pattern (cf. **a′**). Abbreviations as in Fig. 2, and *sp* spinnerets

First, we studied the hatching rates of *Pt*-*FoxB* knockdown embryos after parental RNAi versus control embryos. We found that the hatching rate decreased significantly to < 1% in *FoxB* knockdown embryos, while the hatching rate of approximately 80–90% in control embryos is comparable to that for non-injected wild-type females, cf., e.g., hatching rates in McGregor et al. [54], Königsmann et al. [55]. These first results strongly implied that there is an RNAi-specific effect after injection of *Pt*-*FoxB* dsRNA. We analysed the few hatchlings (first instars) and found that many of these had mildly malformed appendages (Fig. 4). Some of the hatchlings were not able to moult successfully and remained stuck with the distal end of their limbs in the old cuticle.

**Fig. 5:**
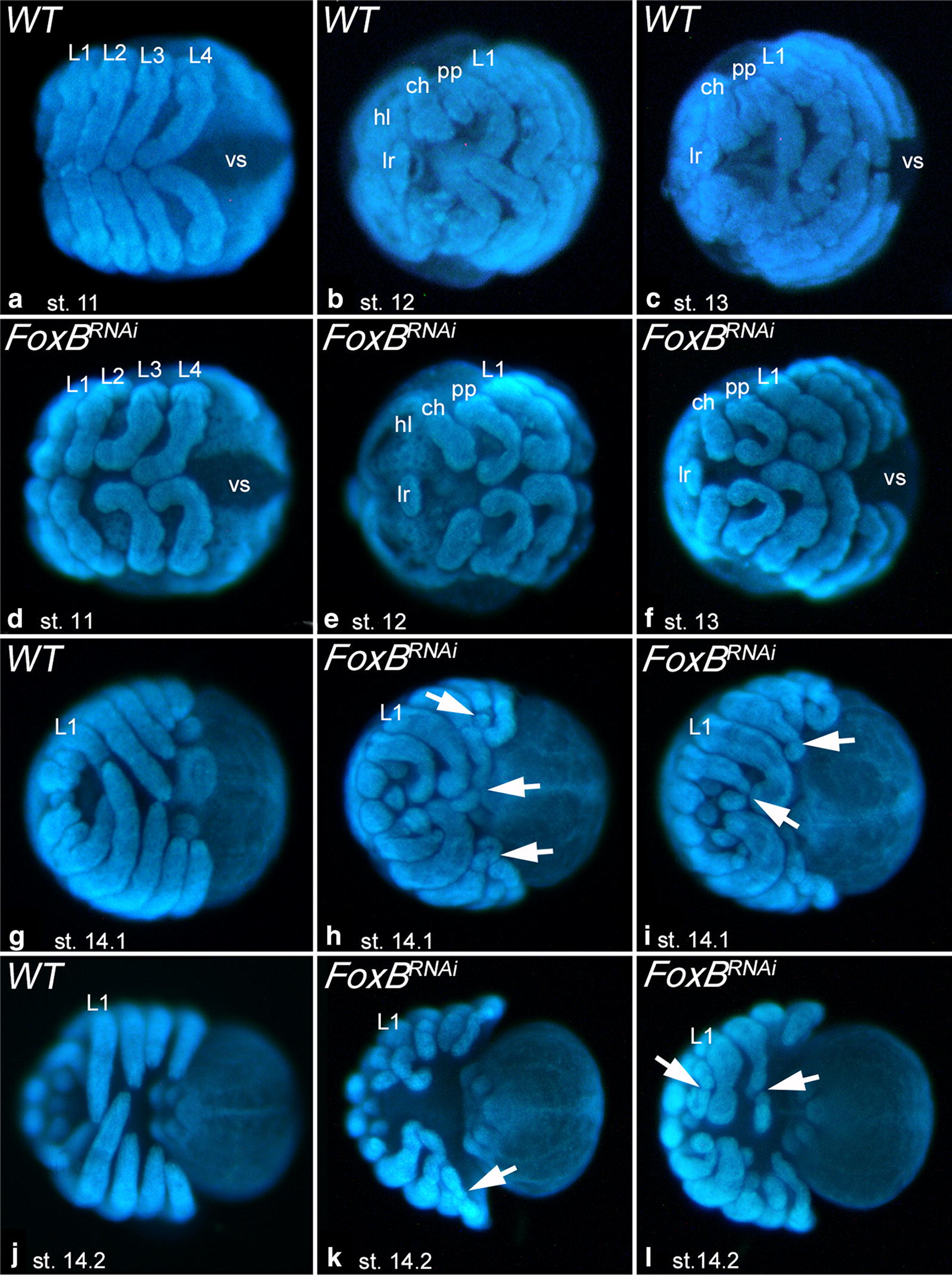
Morphological disturbances in *FoxB* knockdown embryos. Class-I phenotype “Bandyklubba”. **a**–**c** Show wild-type embryos. **d**–**l** Show *FoxB* knockdown embryos. Note the crooked “Bandyklubba”-shaped legs and pedipalps and the budding off of distal limb segments in later stage embryos (arrows). In all panels, anterior is to the left, ventral views. Abbreviations as in Fig. 2 and *hl* head lobe, *vs* ventral sulcus

We then investigated the morphology of embryos of all recovered cocoons of both, RNA-injected females, and buffer-injected females. We discovered four different classes of embryonic phenotypes after *Pt*-*FoxB* knockdown. Quantification of the observed phenotypes is summarized in Additional file 3: Figure S1. Class-I is represented by abnormally developing limbs (Fig. 5). This represents the mildest observed phenotype, and it is most obvious in the relatively long pedipalps and legs, while it is less clearly visible (if at all) in the relatively short chelicerae, the labrum and the opisthosomal appendages. From approximately stage 8.2 onwards, it becomes obvious that the developing pedipalps and legs are broadened compared to wild-type appendages of embryos at comparable developmental stages; at stage 11 this is already very obvious (Fig. 5a, d). As these appendages proceed to develop, they start bending in a peculiar way resembling the shape of a “Bandyklubba” (Swedish, a term that describes the stick used to play the Nordic version of ice hockey) (Fig. 5b, e). Compared with the better known North American ice hockey stick, this stick is shorter and its distal end is more bent, hence the name *Bandyklubba* for this leg phenotype (Fig. 5c, f). At late embryonic stages, these limbs begin to disintegrate in a way that distal region(s) bud off from the proximal region of the legs and pedipalps (Fig. 5g–l). Legs and pedipalps of knockdown embryos are broader than comparable wild-type structures. In Class-I knockdown embryos, the overall morphology is not significantly altered by the appendage function of *Pt*-*FoxB.*

Class-II embryos show an unusually slim germ band. Class-III embryos develop a partially duplicated germ band. In Class-IV embryos, the germ disc does not form correctly. In many of these embryos, the disc is either fully malformed or disintegrated. The yolk is exposed as if the overlying so-called extraembryonic ectoderm did either not form, or disintegrate after formation.

Completely undeveloped eggs likely represent unfertilized eggs that never developed a protective vitelline membrane, and therefore appear as hardened yolk masses after fixation. Our analysis shows that the number of dead embryos is similar in knockdown embryos and wild-type (or control) embryos (Additional file 3: Figure S1).

We also recognized a small percentage of Class-II and Class-IV phenotypes in control embryos, indicating that delayed and small embryos as well as disintegrating (or not-forming) germ discs occasionally occur under natural conditions as well (Additional file 3: Figure S1). This shows that the transition from radial to bilateral symmetry and the onset of germ band formation are critical steps in spider development. However, we did not find a single Class-I or Class-III embryo in control embryos (Additional file 3: Figure S1).

In *FoxB* knockdown cocoons, the number of embryos identified as wild type after fixation at different embryonic stages is higher than the hatching rate suggesting that *FoxB* knockdown may lead to additional effects that are difficult to recognize (e.g., defects of the nervous system).

### Expression of DV appendage patterning genes in *FoxB* knockdown embryos

**Fig. 6:**
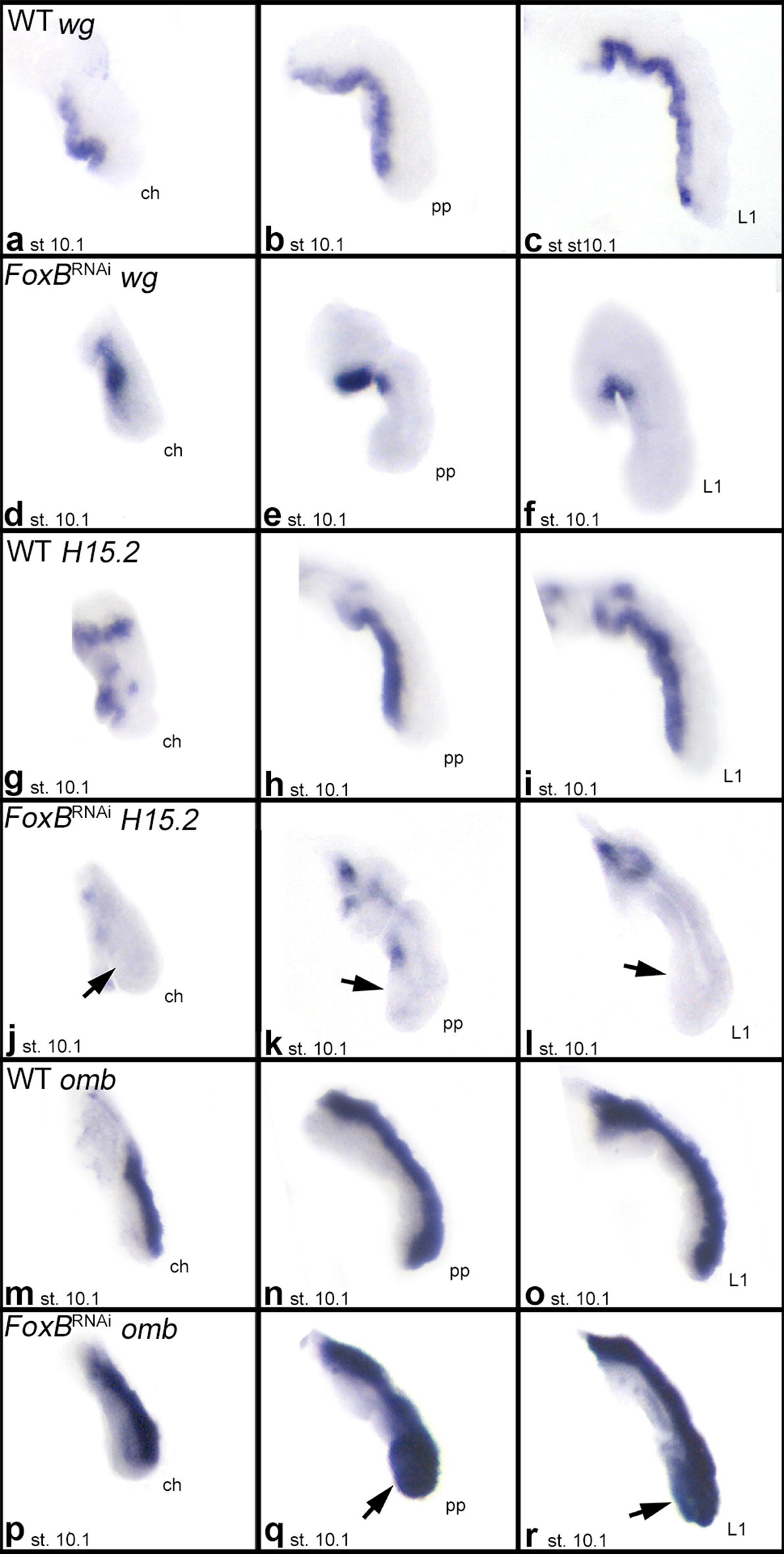
Expression of *wingless* (*wg*) (**a**–**f**), *H15.2* (**g**–**l**) and *optomotor*-*blind* (*omb*) (**m**–**r**) in wild type and *FoxB* knockdown appendages. Class-I phenotype “Bandyklubba”. All panels represent ventral views. Arrows point to areas of altered gene expression in knockdown appendages. Note that *wg* and *H15.2* are expressed along the ventral side of all appendage types in wild-type embryos, and that this expression is lacking in *FoxB* knockdown appendages. The dorsally expressed gene *omb* is expressed in ventral regions in knockdown appendages. Abbreviations as in Fig. 2

In Class-I embryos, legs and pedipalps are abnormally crooked. Since we observed strong expression of *FoxB* along the ventral side of these appendages in the wild type, we tested if DV patterning of the appendages is disturbed after *Pt*-*FoxB* knockdown by analysing the expression patterns of ventral and dorsal marker genes in these embryos. We found that the expression of the conserved arthropod ventral appendage markers *Pt*-*wg*/*Wnt1* (e.g., [8, 10, 38]) and *Pt*-*H15.2* (e.g., [10, 26, 27, 31]) was significantly altered in Class-I appendages (Fig. 6a–l and Additional file 9: Figure S7 and Additional file 10: Figure S8). *Pt*-*wg*/*Wnt1* is absent from the ventral tissue of the appendages except for the most proximal tissue (Fig. 6d–f, Additional file 9: Figure S7). This indicates that *FoxB* is differently required for *wg* expression in the distinct ventral regions along the proximal–distal (PD) axis of the limbs. Similarly, the expression of *H15.2* is absent from ventral tissue in Class-I appendages (Fig. 6j–l, Additional file 10: Figure S8).

The conserved arthropod dorsal limb marker *optomotor*-*blind* (*omb*) (e.g. [10, 27, 31] is still strongly expressed in the dorsal tissue of the limbs in *Pt*-*FoxB* knockdown embryos (Fig. 6p–r, Additional file 11: Figure S9). Interestingly, however, we observed an extension of the *omb* expression pattern into ventral tissue, that normally does not express *Pt*-*omb* (Fig. 6p–r), indicating a partial dorsalisation of the appendages after loss of *Pt*-*FoxB* function. This is predominantly (if not exclusively) observed for the distal region of the pedipalps and legs.

**Fig. 7:**
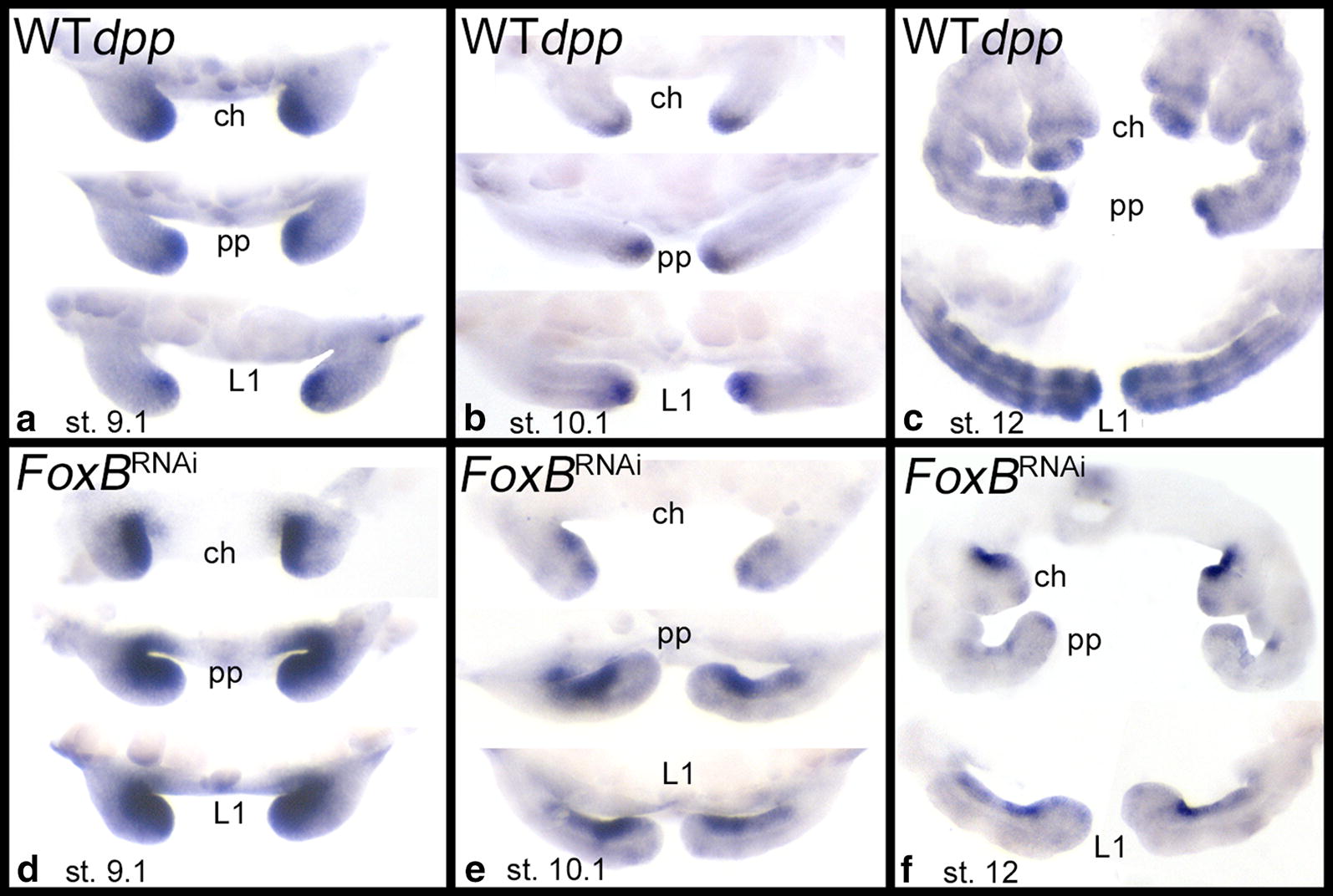
Expression of *Decapentaplegic* (*dpp*) in wild type (WT) (**a**–**c**) and *FoxB* knockdown appendages (**d**–**f**). Class-I phenotype “Bandyklubba”. Note the appearance of *dpp* expression along the ventral region of all appendages after *FoxB* dsRNA treatment. Abbreviations as in Fig. 2

The gene *Decapentaplegic* (*dpp)* encodes a dorsal morphogen acting upstream of *omb* in *Drosophila* [20, 29]. In wild-type *P. tepidariorum* embryos, *Pt*-*dpp* is initially expressed in the tips of the prosomal appendages (Fig. 7a, b), and is later expressed in a striped fashion in the pedipalps and legs (Fig. 7c, Additional file 12: Figure S10). Intriguingly, this pattern is disrupted in *FoxB* knockdown embryos and is replaced by an entirely new expression pattern (Fig. 7d–f, Additional file 12: Figure S10). In the early stages of limb development, *Pt*-*dpp* is expressed along the ventral side of the appendages; initially this includes the ventral portion of the distal tip (Fig. 7d), but subsequently *Pt*-*dpp* is not at all expressed in the distal tip (Fig. 7e, f). The ringed *Pt*-*dpp* pattern that develops in the wild type at approximately stage 12 (Fig. 7c) does not emerge in *Pt*-*FoxB* knockdown embryos.

**Fig. 8:**
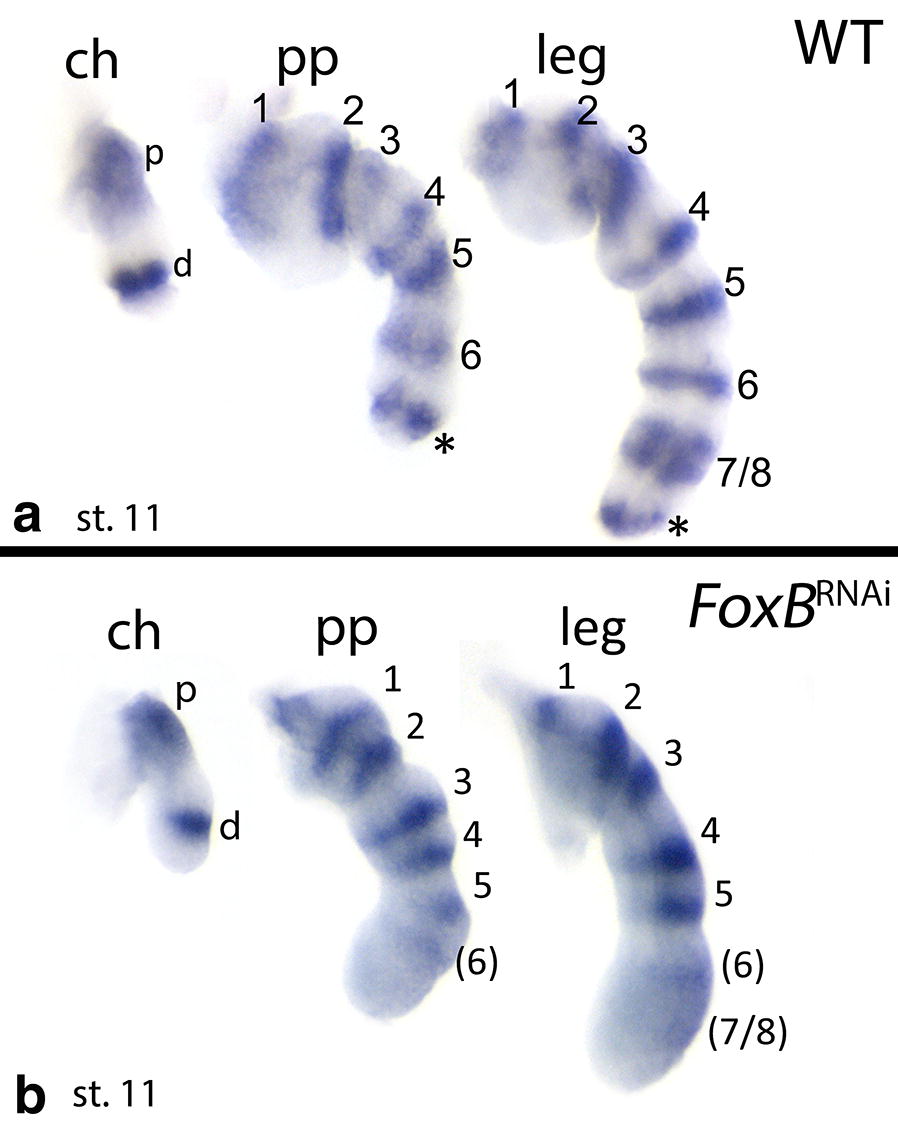
Expression of *odd*-*skipped* (*odd*) in dissected limbs of WT embryos (**a**) and *FoxB* knockdown embryos (**b**). Class-I phenotype “Bandyklubba”. Lateral views. Numbers indicate domains of *odd* expression from proximal to distal. Numbers in brackets indicate weak expression after *FoxB* dsRNA treatment. Black asterisks indicate the most distal domain of *odd*. Note the much weaker or missing ventral expression of *odd* in *FoxB* knockdown embryos. The *odd* gene is likely involved in joint formation, and these results suggest a connection between *FoxB* function and appendage podomerization. Abbreviations as in Fig. 2; *d* distal; *p* proximal

Since the wild-type expression pattern of *FoxB* includes reiterated patterns in the spider limbs which is likely correlated with the position of the developing joints, we investigated the expression of the spider joint marker *odd*-*skipped* (*odd*) [56, 57] in Class-I embryos. In wild-type embryos, *Pt*-*odd* is expressed in nine rings in late developmental stages in the legs, in seven rings in the pedipalps, and two rings in the chelicerae (Fig. 8a). In Class-I limbs, the most distal rings do not form, or expression is very weak (Fig. 8b). Also, in all Class-I appendages, expression disappears from ventral tissue (or is strongly reduced ventrally) (Fig. 8b).

## Discussion

### *FoxB* genes represent evolutionary conserved markers of ventral limb tissue in ventral appendages

We could show that *FoxB* genes are expressed along the ventral side of all ventral appendages and that this expression is conserved in species of diverse panarthropod groups, namely the fly *Drosophila*, the beetle *Tribolium*, the millipede *Glomeris*, the spider *Parasteatoda*, and the onychophoran *Euperipatoides*. This suggests a conserved role for *FoxB* in DV appendage patterning in the entire clade Panarthropoda. Dorsal appendages, like the wings and halteres in *Drosophila*, in contrast, do not express *FoxB,* indicating that its function is restricted to ventral appendages. In all ventral appendage types including the highly modified spider opisthosomal appendages (i.e., the book lungs, the tracheal system, and the spinnerets), *FoxB* is expressed along the ventral ectoderm. This pattern is very similar to that of *wg* and *H15*, two other highly conserved ventral limb marker genes [5, 10, 27, 31].

Exceptions from this rule are the conserved *FoxB* expression domains in the dorsal tissue of the labrum in *Tribolium*, *Glomeris* and *Parasteatoda* (note that we did not investigate expression of *FoxB* in the labral discs of *Drosophila*). This apparent discrepancy, however, can be explained by the hypothesis that the labrum is the result of rotation and fusion of a pair of limbs. As a consequence, ventral and dorsal tissue is reversed in the labrum [52]. The second exception concerns the frontal appendages of the onychophoran which do not express *FoxB*. These appendages are innervated from the protocerebrum and likely are homologous with the arthropod labrum ([58, 59]; discussed in e.g., [60]), although expression of *FoxB* does not support this notion.

### Knockdown of *FoxB* in the spider *Parasteatoda* reveals a specific role in DV appendage development

The highly conserved expression of *FoxB* in the limbs of arthropods and the onychophoran strongly suggests an important and evolutionarily conserved function in panarthropod DV limb development. The functional analysis of *FoxB* in the spider *Parasteatoda tepidariorum* indeed revealed that *FoxB* is required for proper DV patterning during limb axis formation. The Class-I phenotype shows an abnormally crooked distal region of pedipalps and legs, most probably explained by a reduction of ventral tissue. Class-I appendages are also broader and softer than wild-type appendages, indicating that the overall integrity of the limbs is disturbed. This becomes even more evident in later stage Class-I appendages which are characterized by the occurrence of abnormal constrictions that finally lead to the complete budding off of limb parts, especially in the distal region (Fig. 5).

A very similar phenotype has been reported for *wg*/*Wnt1* and its receptor-encoding gene *frizzled*-*1* (*fz1*) in the beetle *Tribolium* [7, 61]. Here the phenotypes are called “candy cane” and “nonpareille”/“pearls on a chain” referring to the bending of the limbs (“candy cane”) and the budding off and fusion of distal limb segments (“nonpareille” and “pearls on a chain”) [7]. *Tribolium fz1* is expressed ubiquitously, but it fulfils a specific function in limb development as revealed by *fz1* knockdown [61]. Although the function of *fz1* is not yet studied in other arthropods, it is also ubiquitously expressed in *Parasteatoda*, allowing for a conserved interaction of Wg and Fz1 in spider limb development [62].

The effect of knockdown of *Pt*-*FoxB* and *Tc*-*wg*, both of which are expressed in conserved patterns along the ventral side of appendages in all hitherto investigated arthropods, is strikingly similar (“Bandyklubba” phenotype and “candy cane” phenotype, respectively) suggesting that they might work in the same conserved gene regulatory network (GRN) in DV limb patterning.

### *FoxB* acts as a key factor in the gene regulatory network (GRN) controlling DV appendage patterning

**Fig. 9:**
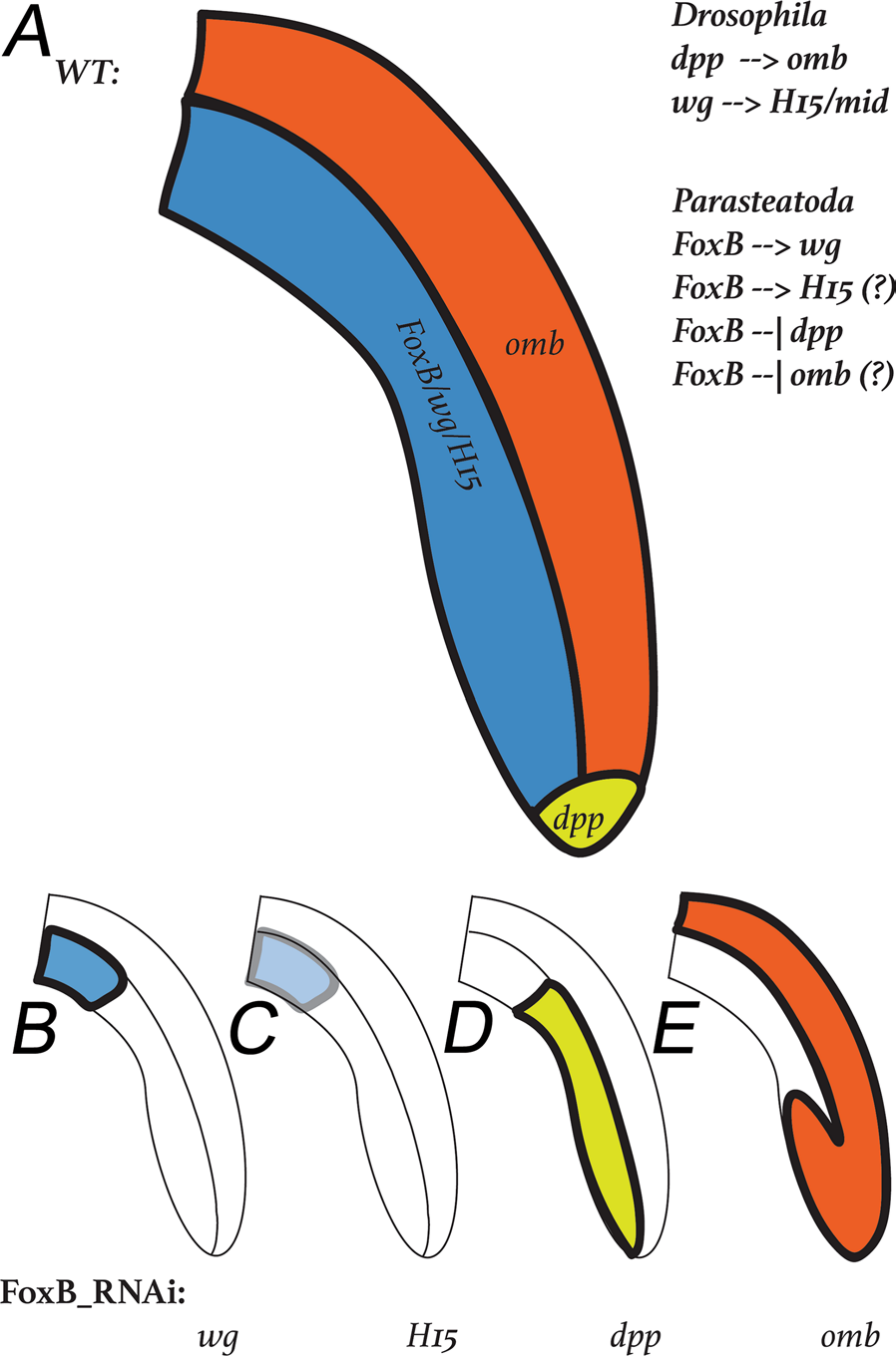
Summary of gene expression in WT legs and FoxB-RNAi knockdown legs. Regulatory interactions of the investigated genes as known from *Drosophila* and as suggested by our results from the spider are indicated. Question marks indicate that it is unclear if the suggested activation of *H15* and repression of *omb* are direct effects of *FoxB*, or whether they represent indirect effects

In the model system *Drosophila melanogaster*, the DV limb axis is determined by the action of the dorsal and ventral morphogens Dpp and Wg, respectively. While *dpp* is specifically expressed in the dorsal sector of the limb imaginal discs, *wg* is specifically expressed in ventral tissue. The expression of the dorsal morphogen encoding gene *dpp* is different in the outgrowing appendages of arthropods with direct development, i.e., the vast majority of all arthropods. Instead of being expressed along the dorsal surface of the limbs, its expression is restricted to the tip (Fig. 9a). Despite these significant differences in gene expression, the so-called topology model has been proposed, that argues for a conserved function of Dpp as dorsal morphogen in a three-dimensional system as represented by directly developing limbs compared to the rather two-dimensional system as represented by the imaginal discs of *Drosophila* [10, 33]. The T-Box transcription factor *optomotor*-*blind* (*omb*) acts downstream of *dpp* in *Drosophila* and is expressed in the dorsal region of the leg imaginal discs (e.g., [63]) (Fig. 9a). This dorsal *omb* expression along the developing limbs was previously shown to be conserved in Panarthropoda [10, 26, 31, 32]. The ventral morphogen encoding gene *wg* is expressed in the ventral sector of the *Drosophila* leg imaginal disc, and its expression in other arthropods is highly conserved as well (e.g., [7, 10, 30]) (Fig. 9a). Downstream of *wg* functions another T-Box transcription factor-encoding gene, *H15* (aka *midline*). Like *wg*, at least one of the *H15* paralogs in each arthropod species is expressed along the ventral side of the outgrowing appendages (e.g., [10, 26, 31]) (Fig. 9a). In summary, the available data are compatible with the notion that the role of *dpp* and *omb* in specifying the dorsal side, as well as the role of *wg* and *H15* in specifying the ventral side are evolutionarily conserved in panarthropods.

After *FoxB* knockdown in the spider, expression of both ventral marker genes, *wg* and *H15.2*, is missing (Fig. 9b, c). This indicates that *FoxB* acts upstream of *wg* in the GRN required for DV patterning. Since *wg* is acting upstream of *H15* in *Drosophila*, the lack of *H15.2* in *FoxB* knockdown appendages could be the result of the lack of *wg*, and thus a secondary effect of *FoxB*, or it could (as assumed for *wg*) be under direct control of *FoxB.* Our experimental setup cannot distinguish between these two possibilities, but it would be interesting to study in future experiments.

The expansion of *dpp* expression along the ventral region in limbs after *FoxB* knockdown indicates that FoxB normally acts as a repressor of *dpp* in ventral tissue, either directly, or via *wg* and/or *H15.2* (Fig. 9d). We note, however, that no aspect of the topology model predicts our observation that the expression of *Pt*-*dpp* is progressively removed from the distal tip in *Pt*-*FoxB* knockdown embryos (Fig. 9d), and therefore this effect of *Pt*-*FoxB* RNAi cannot be explained by the model.

Also, the dorsal factor *omb* is intruding ventral and distal areas of appendages in *FoxB* knockdown embryos, which suggests that FoxB acts directly (or indirectly via Wg and H15.2) as a repressor of *omb* (Fig. 9e). The assumption that Dpp could act as a direct activator of *omb* [10] is not supported by our data, because the expansion of *dpp* along the ventral side of the limbs apparently does not cause ectopic expression of *omb* in this tissue (Fig. 9e). However, it is also possible that ventral tissue is not competent for *omb* expression, even in the presence of *dpp*.

In *Drosophila*, Hedgehog (Hh) activates *dpp* and *wg* in the leg disc due to an early asymmetry that allows ventral and dorsal cells to respond differently to Hh signalling (in dorsal tissue, *dpp* is activated, and in ventral tissue, *wg* is activated). Such asymmetry is provided by the relative earlier expression of Wg in ventral tissue [2, 20, 23]. Consequently, in the absence of Wg, Hh would activate *dpp* in ventral tissue, instead of *wg* [20].

This scenario is in line with our data. Since *wg* is absent from ventral tissue in *FoxB* knockdown embryos as a result of the missing function of FoxB, now *dpp* is dominantly expressed in this tissue. Once the asymmetry between *wg* and *dpp* expressing tissue is established, Dpp and Wg act as mutual antagonists in the *Drosophila* imaginal discs [20]. If this mutual antagonistic function is conserved, or at least the repressive function of Wg on Dpp, this might explain why *dpp* expands into the now *wg*-free ventral limb tissue after FoxB knockdown. Again, we cannot distinguish between a possible direct or indirect repression of *dpp* via FoxB or/and Wg. Either way, our data suggest that *FoxB* is acting at a high level in the GRN orchestrating DV limb patterning.

### Evidence for different regulatory mechanisms acting along the AP axis of developing limbs

It has been shown that different regions along the AP axis of the *Drosophila* leg are under control of different GRNs, or that given GRNs act differently in different regions of the leg. For example, the most proximal region of the *Drosophila* leg, the coxa, never expresses *Distal*-*less* (*Dll*), a gene that is otherwise involved in the formation of all other podomeres (leg segments) (reviewed in [64]). It has also been shown that *wg* plays a specific role in the development of the coxa [65]. Similarly, it also appears that the proximal region (including the coxa) is patterned differently in the beetle *Tribolium*. Interestingly, here *wg* appears to have the opposite effect. While distal regions of the legs are affected in *wg* knockdown and *Fz1* knockdown embryos, this is not the case for the coxal region [7, 61]. Therefore, it is possible that the proximal region and the distal region (defined as distal to the coxa) are generally regulated differently in arthropods [66, 67].

Our results on *Parasteatoda FoxB* function support this hypothesis and suggest that the differences between proximal and distal leg development may indeed date back to the last common ancestor of insects and spiders, i.e., the arthropod ancestor. Although *Pt*-*FoxB* is expressed all along the ventral side of pedipalps and legs, its knockdown affects only the distal and medial, but not the proximal *Pt*-*wg* expression (Fig. 9b). Similarly, *Pt*-*FoxB* knockdown leads to the misexpression of *Pt*-*omb* in the distal portions of pedipalps and legs, while medial and proximal portions are not affected (Fig. 9e). The most intriguing result, however, is the complete change of the *Pt*-*dpp* expression pattern after *Pt*-*FoxB* RNAi, especially in the distal tip. In this case, the loss of *Pt*-*FoxB* influences *Pt*-*dpp* expression even in portions of the limbs that never express *Pt*-*FoxB*. The reason for this is currently not clear.

### Evidence for the coupling of DV patterning and joint formation

In *Drosophila*, the correct formation of joints depends on the PD patterning system and the so-called leg gap genes (e.g. [68, 69]; summarized in [70]). In a combinatorial mode, they activate Delta/Notch signalling (e.g., [71–73]) and downstream of Delta/Notch signalling act, e.g., the *odd*-*skipped* family genes, including *odd*-*skipped* (*odd*) itself [74, 75]. It has been shown that the involvement of Delta/Notch signalling and its downstream factors such as *odd* in arthropod joint formation is conserved in arthropods beyond *Drosophila* [76]. In the spider *Cupiennius salei*, the *odd* ortholog *odd*-*related*-*1* (one of three identified odd-related genes in this spider) is expressed in concentric rings in the limbs downstream of Delta/Notch signalling and its function is clearly correlated with that of joint formation [76]. The same expression pattern is seen for *odd* in the limbs of *Parasteatoda* (Fig. 8).

Remarkably, we find that *odd* expression in concentric rings is disturbed after knocking down *FoxB*, but only in the ventral sector, while expression along the dorsal side of the limbs is not affected (except for the distal region where expression of *odd* is completely lost) (Fig. 8). Double in situ revealed that *odd* is indeed co-expressed with the patches of enhanced expression of *FoxB* (Fig. 3k). Together, this implies that *odd* expression in the limbs is likely under control of the DV patterning system downstream of FoxB function, at least ventrally. Since *odd* is one of the genes that is involved in joint formation in spiders, this finding is the first potential evidence that joint formation and DV patterning may be connected.

## Supplementary information

**Additional file 1: Table S1.** Primer sequences.

**Additional file 2: Table S2.** Accession numbers.

**Additional file 3: Figure S1.** Summary of phenotypes found in *FoxB* knockdown embryos compared to control embryos. The total numbers of investigated control and *FoxB* knockdown embryos is indicated on top of the bars in the diagram. The table gives a quantification of the observed phenotypes in relation to control embryos that show mostly wild-type phenotypes. Legend explanation: WT, wild type; Class-I, “Bandyklubba” phenotype (crooked limbs); Class-II, embryos with small germ bands; Class-III, partially duplicated germ band; Class-IV, germ band not forming (irregular germ disc); dead, unfertilized/not developing.

**Additional file 4: Figure S2.** Phylogenetic analysis. Bayesian phylogeny of forkhead domain amino acid sequences of FoxA, FoxB and FoxC genes of *Drosophila melanogaster* (Dm), *Tribolium castaneum* (Tc), *Glomeris marginata* (Gm), and *Parasteatoda tepidariorum* (Pt). *Drosophila* FoxQ2 serves as outgroup. Branch support (posterior probabilities) is given for each main branch. This simple analysis shows that *FoxB* orthologs can easily be distinguished from other closely related Fox genes such as *FoxA* and *FoxC*.

**Additional file 5: Figure S3.** Expression of *Drosophila melanogaster FoxB2* in leg discs, the antennal disc, and the eye disc. Note that the expression is identical to that of *FoxB1*. Like *FoxB1*, *FoxB2* is not expressed in the wing discs and the haltere discs (not shown).

**Additional file 6: Figure S4.** Expression of *Tribolium FoxB1* in legs of embryos of different developmental stages. Note that the prepared appendages are still connected showing that expression is indeed along their ventral side. Abbreviations: d, dorsal side; v, ventral side.

**Additional file 7: Figure S5.** Expression of *Glomeris marginata FoxB*. In all panels, anterior is to the left, ventral views. Developmental stages are indicated. Except for expression in the appendages (see main text), *Glomeris FoxB* is also expressed in the ventral nervous system (arrows) and the anal valves. Abbreviations: an, antenna; av, anal valves; lr, labrum; mx, maxilla; T1, first trunk segment.

**Additional file 8: Figure S6.** Expression of *Tribolium castaneum FoxB2*. In all panels, expression in to the left, ventral views. Note that the expression of *Tribolium FoxB1* (main text) and *FoxB2* is identical. Arrows point to expression in the ventral nervous system. Abbreviations: an, antenna; T1, first thoracic segment.

**Additional file 9: Figure S7.** Expression of *wingless* (*wg*) in wild type (A–C) and *FoxB* knockdown embryos (D–F). In all panels, anterior is to the left, ventral views. Embryos shown in panels A–C and D–F represent different views on the same embryo. Note the reduced/lacking expression in the appendages. Abbreviations as in Fig. 2; hl, head lobe.

**Additional file 10: Figure S8**. Expression of *H15.2* in wild type (A–D) and *FoxB* knockdown embryos (E–H). In all panels, anterior is to the left, ventral views (except panel D, lateral view). Arrows point to missing expression in ventral tissue of the legs and pedipalps. Embryos shown in panels A–D and E–H represent different views on the same embryo. Abbreviations as in Fig. 2; hl, head lobe; vs, ventral sulcus.

**Additional file 11: Figure S9.** Expression of *optomotor-blind* (*omb*) in wild type (A–D) and *FoxB* knockdown embryos (E–H). In all panels, anterior is to the left, ventral views (except panel D, lateral view). Arrows point to ectopic expression of *omb* in the pedipalps and legs, but not the chelicerae (arrowhead). Embryos shown in panels A–D and E–H represent different views on the same embryo. Abbreviations as in Fig. 2; hl, head lobe.

**Additional file 12: Figure S10.** Expression of *Decapentaplegic* (*dpp*) in wild type (A–C) and *FoxB* knockdown embryos (D–F). In all panels, anterior is to the left, ventral views. Abbreviations as in Fig. 2.
