# Supplementary material for: FoxB, a new and highly conserved key factor in arthropod dorsal–ventral (DV) limb patterning
Source: EvoDevo. 2019 Nov 8;10:28. doi: 10.1186/s13227-019-0141-6 (PMC6842170; doi:10.1186/s13227-019-0141-6)
Supplement: Supplementary file 1 — Additional file 1: Table S1. Primer sequences. [file 13227_2019_141_MOESM1_ESM.docx]

| Gene | Forward primer | Backward primer |
| --- | --- | --- |
| *Dm-FoxB1* | GCTGCTCCCGCTTATGTTCCTG | ATACCGCTGCACTCGACCACAT |
| *Dm-FoxB2* | GCCATAATACACTCGCCGCAAA | CGTTATGAAAAAGCGGCGGTGT |
| *Tc-FoxB1* | TACGGGGACCAGAAACCACC | aggtggagggggtcatcatg |
| *Tc-FoxB2* | GGGTGGTGTGGTGTCTGGAA | aatcgagcgcagggccaact |
| *Gm-FoxB* | ATCAGAAACCGCCGTA | gcttgctggtatgggt |
| *Pt-FoxB* | GTATTCCAAAGTGTGTCTTG | gaatcgtccgatacaacatc |
| *Ek-FoxB* | GATCAAAAACCTCCTTATTC | atttgtttaggggagtcc |
| *M13F* | GTAAAACGACGGCCAG | |
| *M13R* | CAGGAAACAGCTATGAC | |
| *T7* | GTAATACGACTCACTATAGGGCGAAT | |
| *T7-SP6* | GTAATACGACTCACTATAGGGATTTAGGTGACACTATAGAATACTCAAGC | |
