# Supplementary material for: FoxB, a new and highly conserved key factor in arthropod dorsal–ventral (DV) limb patterning
Source: EvoDevo. 2019 Nov 8;10:28. doi: 10.1186/s13227-019-0141-6 (PMC6842170; doi:10.1186/s13227-019-0141-6)
Supplement: Supplementary file 2 — Additional file 2: Table S2. Accession numbers. [file 13227_2019_141_MOESM2_ESM.docx]

| Gene | Accession number |
| --- | --- |
| *Dm-FoxB1* | NM_079771.2 |
| *Dm-FoxB2* | NM_079772.2 |
| *Gm-FoxB* | LT992886 |
| *Tc-FoxB1* | XM_015981390.1 |
| *Tc-FoxB2* | XM_962963.3 |
| *Pt-FoxB* | XM_016057196.1 |
| *Ek-FoxB* | LT992887 |
| Additional sequences used for phylogenetic analysis: | |
| *Dm-FoxA* | NP_524542.1 |
| *Gm-FoxA* | CUW78643.1 |
| *Tc-FoxA* | NP_001034503.2 |
| *Pt-FoxA1* | NP_001310762.1 |
| *Pt-FoxA2* | XP_015924819.1 |
| *Ek-FoxA* | CEP25539.1 |
| *Dm-FoxC* | NP_524202.1 |
| *Gm-FoxC* | CAK50838.1 |
| *Tc-FoxC* | XP_001812698.1 |
| *Ek-FoxC* | LR028006 |
| *Dm-FoxQ2* | NP_651951.1s |
