# Supplementary figures and images for: FoxB, a new and highly conserved key factor in arthropod dorsal–ventral (DV) limb patterning
Source: EvoDevo. 2019 Nov 8;10:28. doi: 10.1186/s13227-019-0141-6 (PMC6842170; doi:10.1186/s13227-019-0141-6)

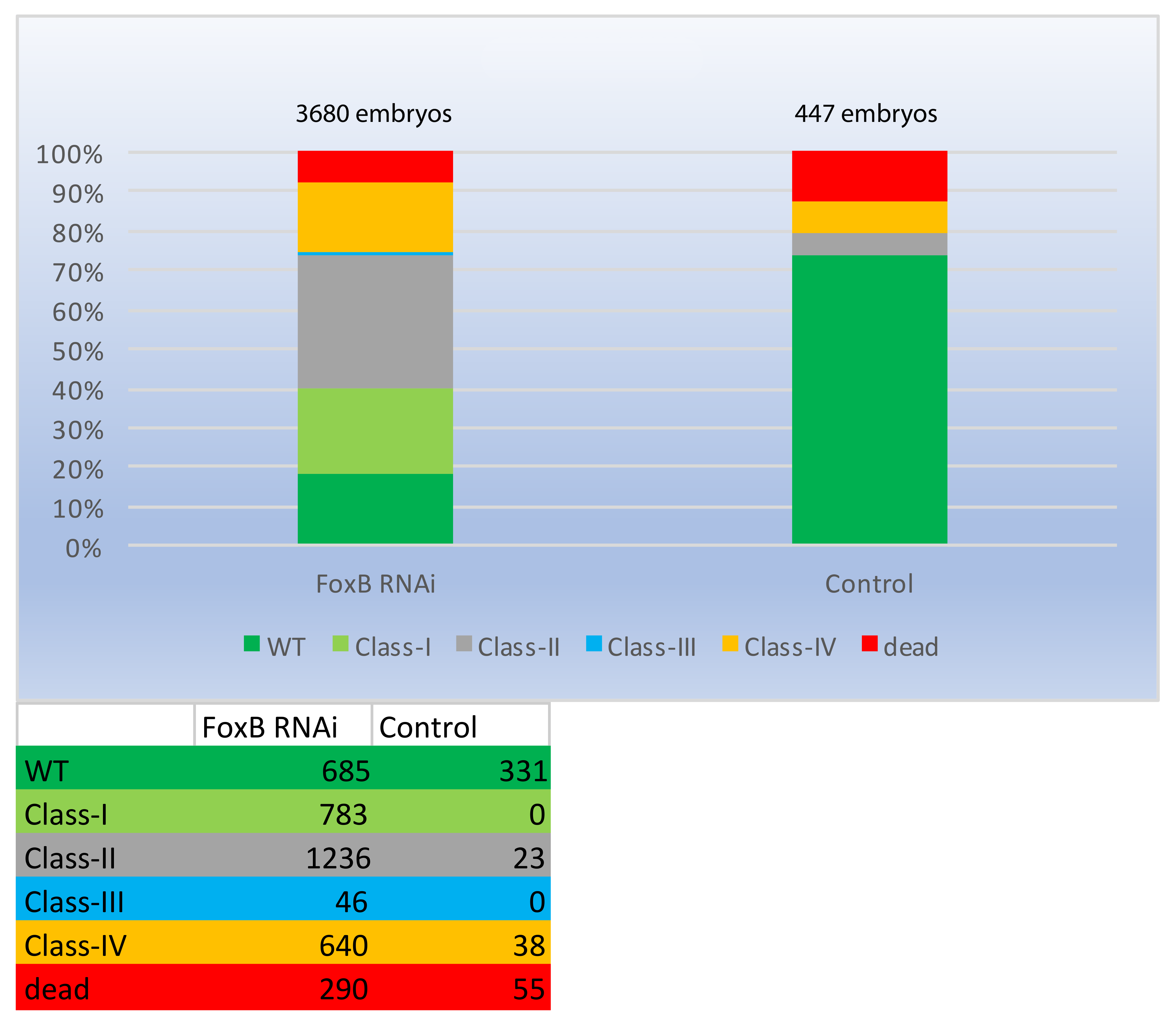

Supplement: Supplementary file 3 — Additional file 3: Figure S1. Summary of phenotypes found in FoxB knockdown embryos compared to control embryos. The total numbers of investigated control and FoxB knockdown embryos is indicated on top of the bars in the diagram. The table gives a quantification of the observed phenotypes in relation to control embryos that show mostly wild-type phenotypes. Legend explanation: WT, wild type; Class-I, “Bandyklubba” phenotype (crooked limbs); Class-II, embryos with small germ bands; Class-III, partially duplicated germ band; Class-IV, germ band not forming (irregular germ disc); dead, unfertilized/not developing. [file 13227_2019_141_MOESM3_ESM.tif]

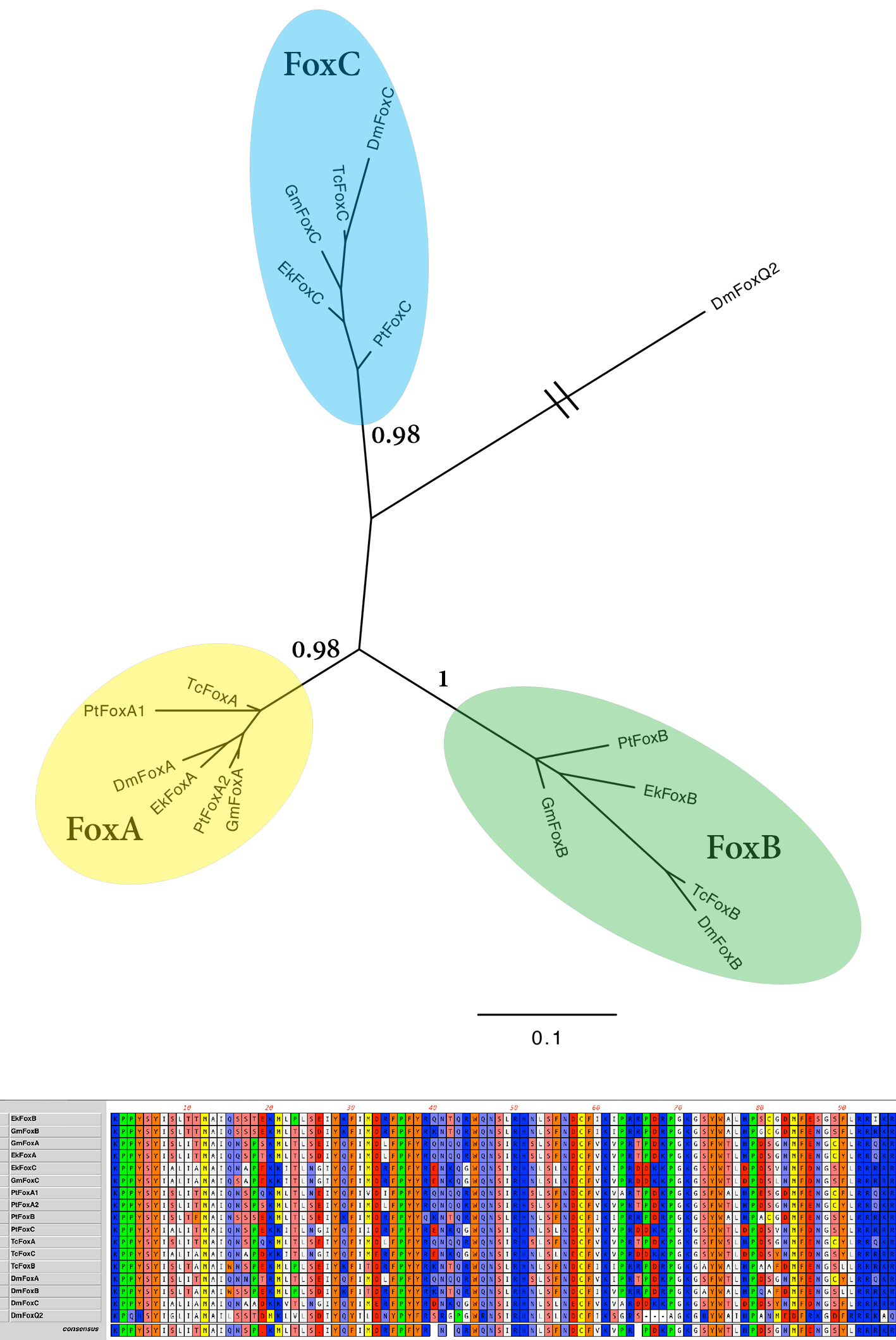

Supplement: Supplementary file 4 — Additional file 4: Figure S2. Phylogenetic analysis. Bayesian phylogeny of forkhead domain amino acid sequences of FoxA, FoxB and FoxC genes of Drosophila melanogaster (Dm), Tribolium castaneum (Tc), Glomeris marginata (Gm), and Parasteatoda tepidariorum (Pt). Drosophila FoxQ2 serves as outgroup. Branch support (posterior probabilities) is given for each main branch. This simple analysis shows that FoxB orthologs can easily be distinguished from other closely related Fox genes such as FoxA and FoxC. [file 13227_2019_141_MOESM4_ESM.tif]

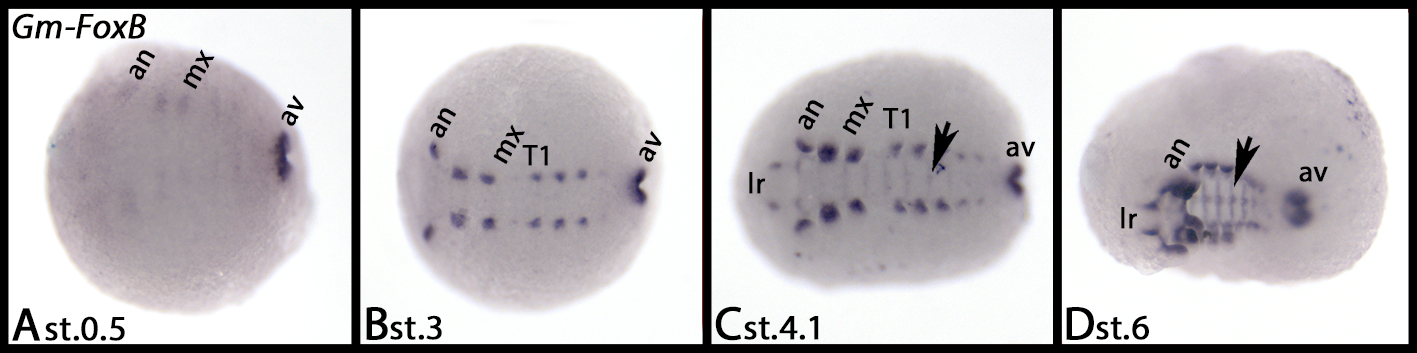

Supplement: Supplementary file 5 — Additional file 5: Figure S3. Expression of Drosophila melanogaster FoxB2 in leg discs, the antennal disc, and the eye disc. Note that the expression is identical to that of FoxB1. Like FoxB1, FoxB2 is not expressed in the wing discs and the haltere discs (not shown). [file 13227_2019_141_MOESM5_ESM.tif]

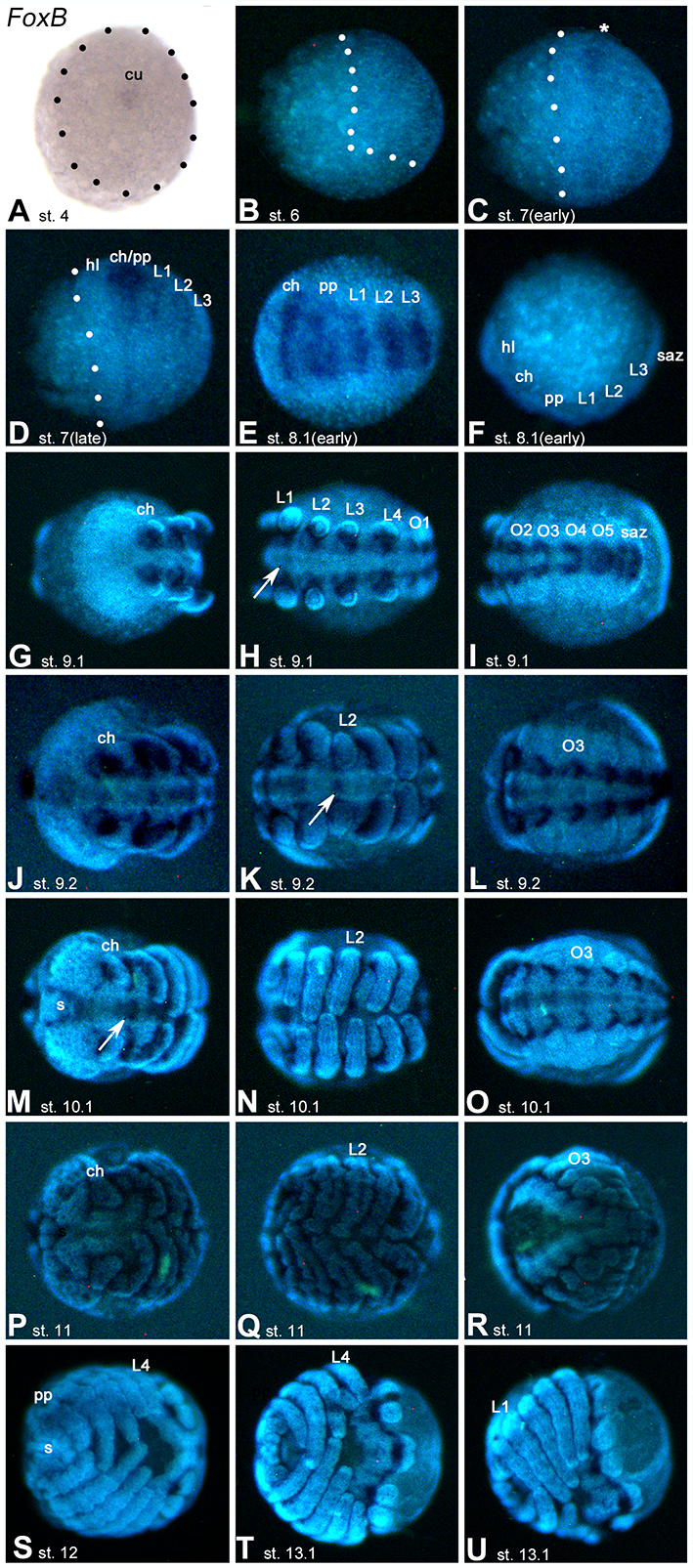

Supplement: Supplementary file 6 — Additional file 6: Figure S4. Expression of Tribolium FoxB1 in legs of embryos of different developmental stages. Note that the prepared appendages are still connected showing that expression is indeed along their ventral side. Abbreviations: d, dorsal side; v, ventral side. [file 13227_2019_141_MOESM6_ESM.tif]

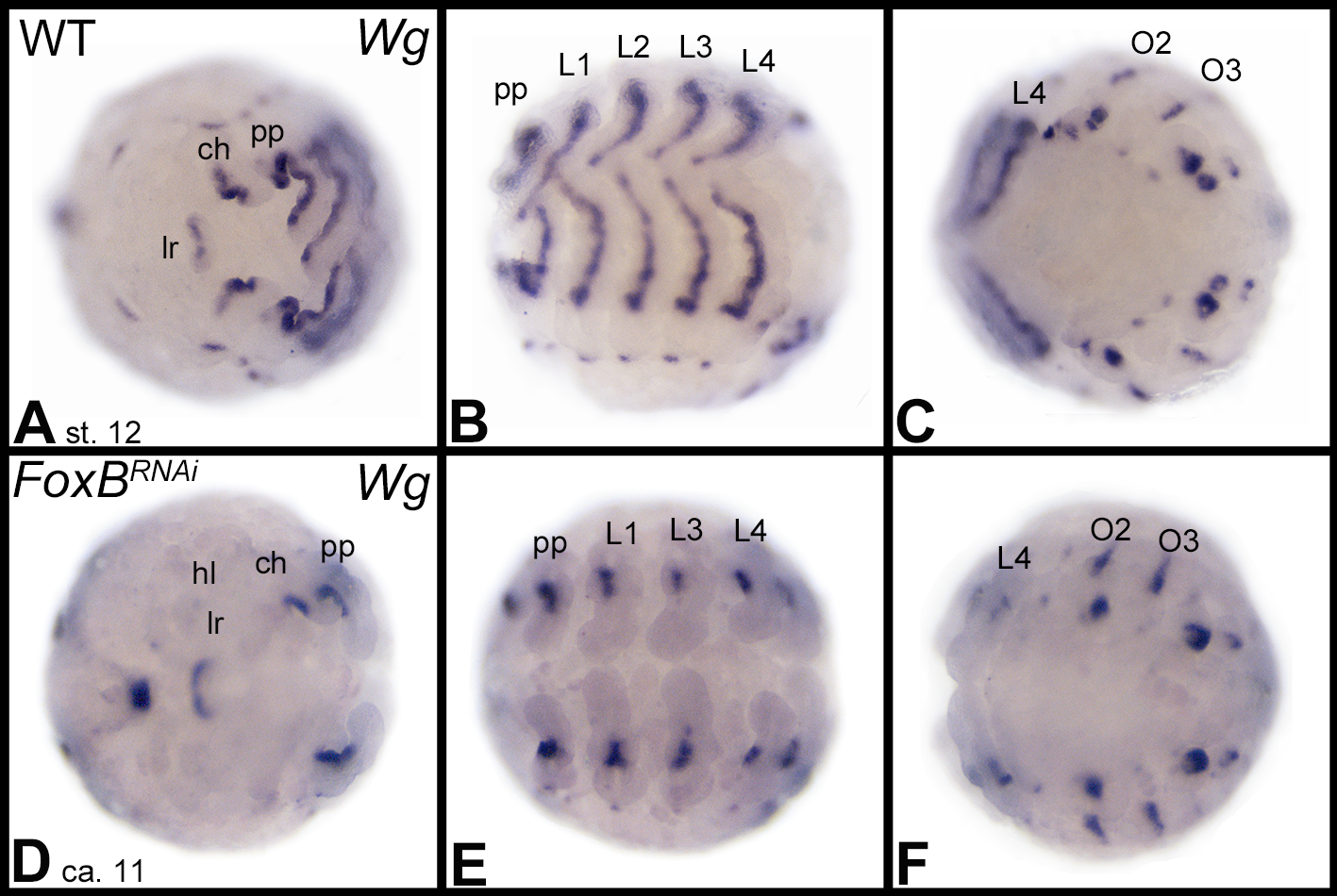

Supplement: Supplementary file 7 — Additional file 7: Figure S5. Expression of Glomeris marginata FoxB. In all panels, anterior is to the left, ventral views. Developmental stages are indicated. Except for expression in the appendages (see main text), Glomeris FoxB is also expressed in the ventral nervous system (arrows) and the anal valves. Abbreviations: an, antenna; av, anal valves; lr, labrum; mx, maxilla; T1, first trunk segment. [file 13227_2019_141_MOESM7_ESM.tif]

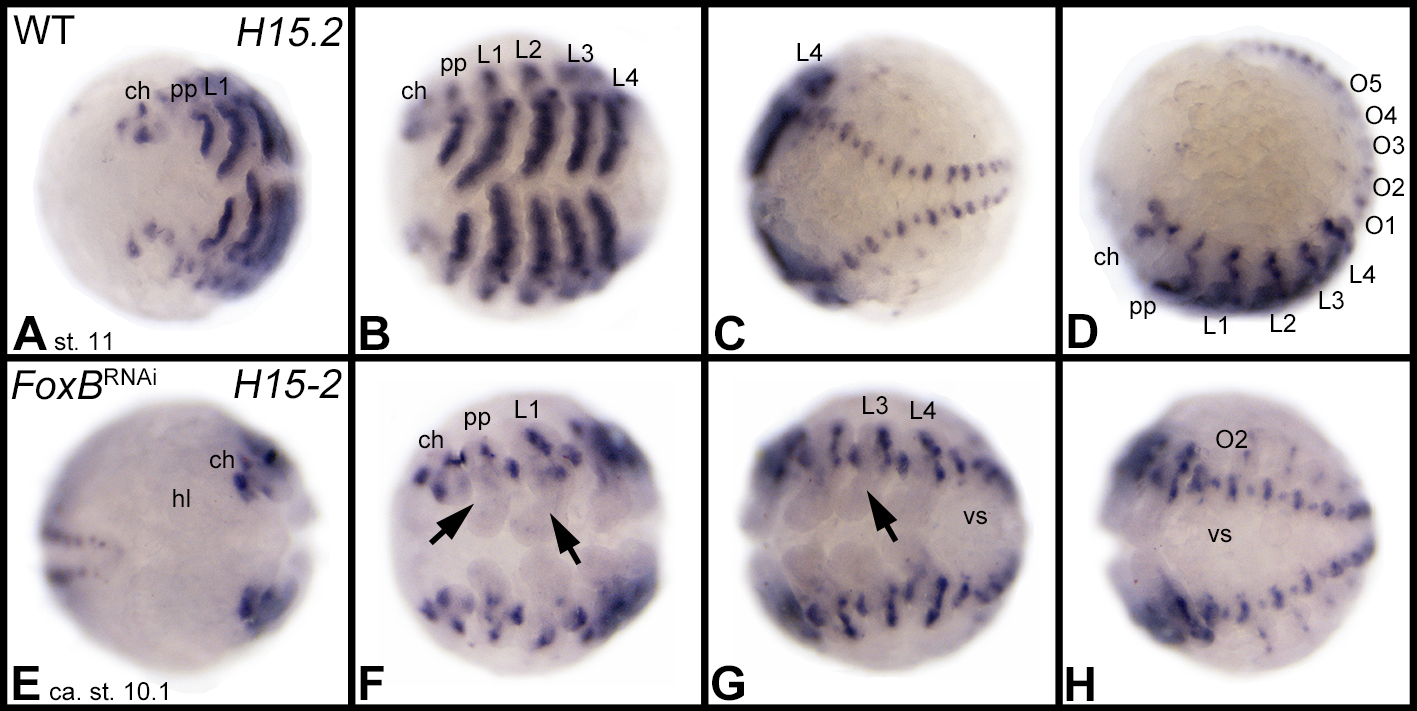

Supplement: Supplementary file 8 — Additional file 8: Figure S6. Expression of Tribolium castaneum FoxB2. In all panels, expression in to the left, ventral views. Note that the expression of Tribolium FoxB1 (main text) and FoxB2 is identical. Arrows point to expression in the ventral nervous system. Abbreviations: an, antenna; T1, first thoracic segment. [file 13227_2019_141_MOESM8_ESM.tif]

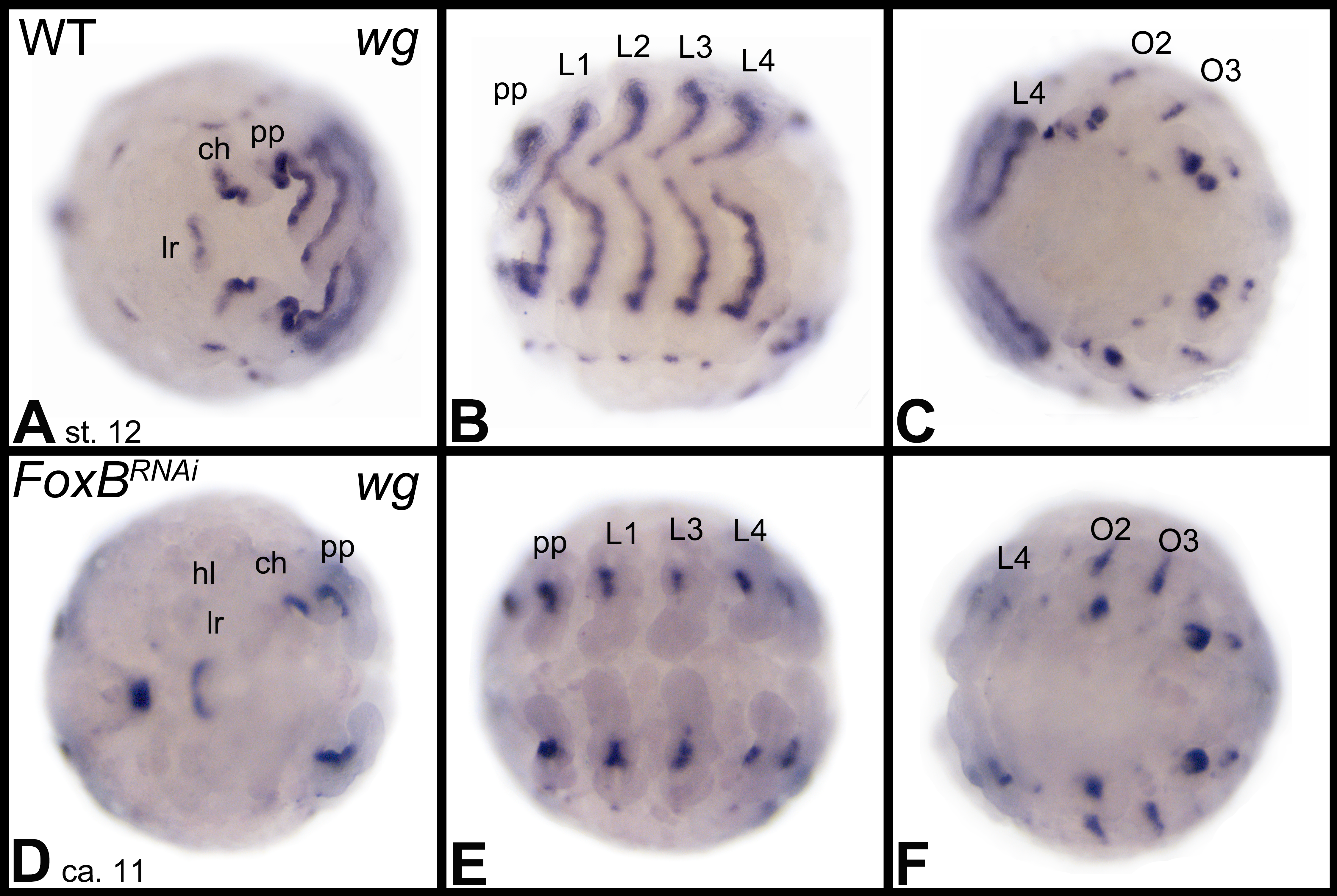

Supplement: Supplementary file 9 — Additional file 9: Figure S7. Expression of wingless (wg) in wild type (A–C) and FoxB knockdown embryos (D–F). In all panels, anterior is to the left, ventral views. Embryos shown in panels A–C and D–F represent different views on the same embryo. Note the reduced/lacking expression in the appendages. Abbreviations as in Fig. 2; hl, head lobe. [file 13227_2019_141_MOESM9_ESM.tif]

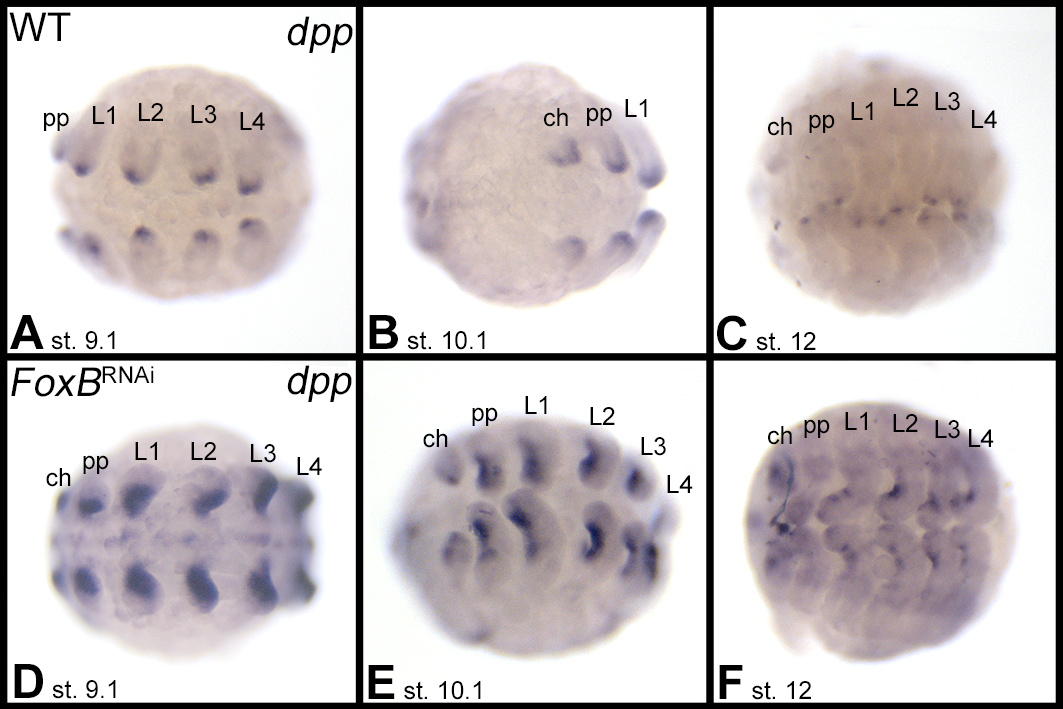

Supplement: Supplementary file 10 — Additional file 10: Figure S8. Expression of H15.2 in wild type (A–D) and FoxB knockdown embryos (E–H). In all panels, anterior is to the left, ventral views (except panel D, lateral view). Arrows point to missing expression in ventral tissue of the legs and pedipalps. Embryos shown in panels A–D and E–H represent different views on the same embryo. Abbreviations as in Fig. 2; hl, head lobe; vs, ventral sulcus. [file 13227_2019_141_MOESM10_ESM.tif]

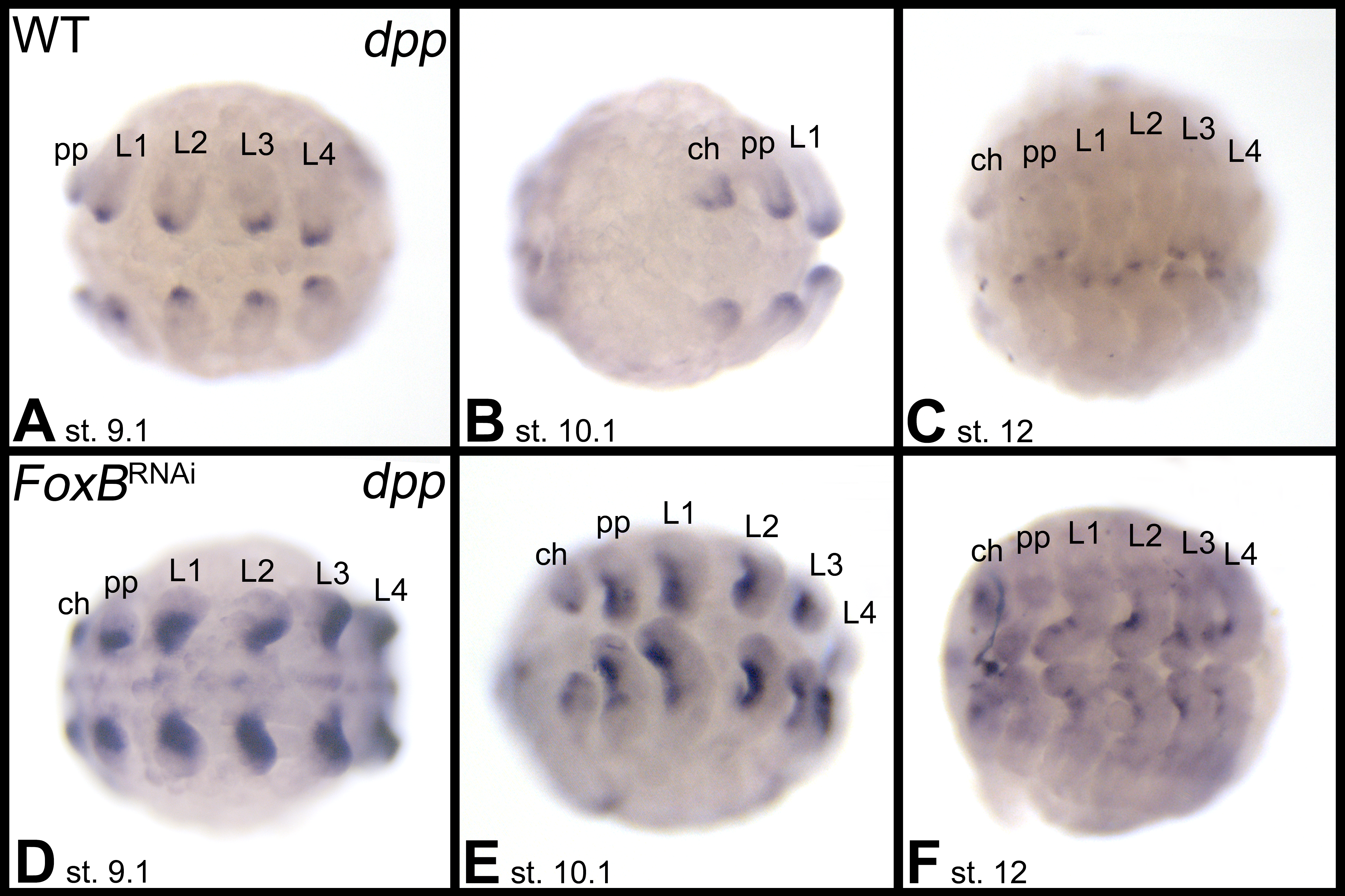

Supplement: Supplementary file 12 — Additional file 12: Figure S10. Expression of Decapentaplegic (dpp) in wild type (A–C) and FoxB knockdown embryos (D–F). In all panels, anterior is to the left, ventral views. Abbreviations as in Fig. 2. [file 13227_2019_141_MOESM12_ESM.tif]
